# Age-related mitochondrial alterations in brain and skeletal muscle of the YAC128 model of Huntington disease

**DOI:** 10.1038/s41514-021-00079-2

**Published:** 2021-10-14

**Authors:** Kristina Bečanović, Muhammad Asghar, Izabella Gadawska, Shiny Sachdeva, David Walker, Eduardo. R. Lazarowski, Sonia Franciosi, Kevin H. J. Park, Hélène C. F. Côté, Blair R. Leavitt

**Affiliations:** 1 Centre for Molecular Medicine and Therapeutics, Department of Medical Genetics, University of British Columbia, Vancouver, BC Canada; 2 Department of Clinical Neuroscience, Karolinska Institutet, Stockholm, Sweden; 3 Department of Medicine, Division of Infectious Diseases, Karolinska Institutet, Stockholm, Sweden; 4 Department of Infectious Diseases, Karolinska University Hospital, Stockholm, Sweden; 5 Department of Pathology & Laboratory Medicine, University of British Columbia, Vancouver, BC Canada; 6 The James Hogg iCAPTURE Centre for Cardiovascular and Pulmonary Disease, St Paul’s Hospital, Vancouver, BC Canada; 7 Cystic Fibrosis Research Center, Marsico Lung Institute, University of North Carolina, Chapel Hill, NC USA; 8 Department of Pediatrics, University of British Columbia, Vancouver, BC Canada; 9 Department of Psychology and Neuroscience Program, Central Michigan University, Mount Pleasant, MI USA

**Keywords:** Ageing, Neurodegeneration

## Abstract

Mitochondrial dysfunction and bioenergetics failure are common pathological hallmarks in Huntington’s disease (HD) and aging. In the present study, we used the YAC128 murine model of HD to examine the effects of mutant huntingtin on mitochondrial parameters related to aging in brain and skeletal muscle. We have conducted a cross-sectional natural history study of mitochondrial DNA changes in the YAC128 mouse. Here, we first show that the mitochondrial volume fraction appears to increase in the axons and dendrite regions adjacent to the striatal neuron cell bodies in old mice. Mitochondrial DNA copy number (mtDNAcn) was used as a proxy measure for mitochondrial biogenesis and function. We observed that the mtDNAcn changes significantly with age and genotype in a tissue-specific manner. We found a positive correlation between aging and the mtDNAcn in striatum and skeletal muscle but not in cortex. Notably, the YAC128 mice had lower mtDNAcn in cortex and skeletal muscle. We further show that mtDNA deletions are present in striatal and skeletal muscle tissue in both young and aged YAC128 and WT mice. Tracking gene expression levels cross-sectionally in mice allowed us to identify contributions of age and genotype to transcriptional variance in mitochondria-related genes. These findings provide insights into the role of mitochondrial dynamics in HD pathogenesis in both brain and skeletal muscle, and suggest that mtDNAcn in skeletal muscle tissue may be a potential biomarker that should be investigated further in human HD.

## Introduction

Mitochondrial dysfunction has been implicated to play a critical role in both aging and as a major contributing factor to the pathogenesis in Huntington’s disease (HD), although this disorder is not directly caused by mitochondrial mutations. HD is an autosomal dominant, typically adult-onset neurodegenerative disease, characterized by progressive motor dysfunction, cognitive decline and psychiatric disturbances including metabolic deficits^1^. The disease is caused by the expansion of CAG trinucleotide repeats in the huntingtin (*HTT)* gene resulting in abnormally long polyglutamine expansions in the huntingtin protein (HTT), which confers toxic functions to the mutant HTT protein (mHTT). One of the most striking pathological hallmarks of HD is the selective degeneration of the basal ganglia, with the medium spiny neurons (MSNs) in the striatum being particularly vulnerable together with the cortical neurons that project to the striatum. The HTT protein is, however, ubiquitously expressed both in the central nervous system and in peripheral tissue including skeletal muscle^2–4^.

Mitochondria are highly dynamic organelles that move along axons and dendrites in neurons, thus playing an important role in the cellular bioenergetics, regulation of Ca^2+^ and redox-signaling, developmental and synaptic plasticity, and as key mediators of cell survival and apoptosis^5^. Mitochondrial dysfunction including defective mitochondrial biogenesis, aberrant mitochondrial dynamics and trafficking^6,7^, autophagy dysfunction^8^ and transcriptional dysregulation are consistent features in HD^9^. However, it is unclear whether many of the observed defects contribute to HD pathogenesis or if they are a consequence of HD pathology. Nevertheless, substantial evidence suggests that mitochondrial dysfunction occurs in the brain and peripheral tissues of individuals with HD and in HD animal models.

Decreased mitochondrial respiratory chain (MRC) complex II, III and IV activities have been reported in HD caudate^10^ and putamen^11^ in postmortem brain^12^. Other supporting evidence include abnormal brain energy metabolism with impaired cortical glucose metabolism at early disease stages and increased lactate levels in a non-specific fashion in the brain of individuals with HD^13,14^. Studies further suggest that mHTT adversely affects the mitochondria by modifying gene transcription of fusion and fission genes, increasing mitochondrial fragmentation and distorting the anterograde and retrograde mitochondrial transport^15–17^. Several studies have reported on skeletal muscle pathology^18^ with evidence for bioenergetic deficits as individuals with HD exhibit reduced muscle strength^19^ and weight loss despite normal/increased food intake^20^, which hypothetically may be caused by impaired mitochondrial ATP synthesis. Muscular atrophy and wasting are commonly observed late in the disease, although case-reports have shown myopathy in pre-symptomatic HD gene carriers^21^. Studies on individuals with HD further showed a deficit in oxidative function in both manifest and pre-manifest subjects^22^ and reduced maximum rate of ATP production in skeletal muscle^23^. Abnormal mitochondria with various ultrastructural changes and potential functional implications have been reported in human HD brain^24^, peripheral cell tissue from HD patients^25^, and in HD transgenic mouse models^17,26^. Relating to mitochondrial DNA (mtDNA) damage in HD, an elevated mutation rate in mtDNA has been proposed due to the high frequency of mtDNA deletions found in HD patient lymphocytes^27^ and in the striatum and cortex of R6/2 HD mice^28^. Studies assessing the so-called common mtDNA deletion (mtDNA4977) have further been contradictory, showing increased levels in HD cortex but comparable levels in HD putamen^29^. Another study showed lower levels of mtDNA4977 in HD cortex and HD putamen and comparable levels in HD caudate compared to controls^30^.

In addition to being a feature of HD, mitochondrial dysfunction is a well-recognized hallmark of cellular aging, where aging is characterized by a decay of mitochondrial function and oxidative phosphorylation (OXPHOS) capacity, concomitant with alterations in mitochondrial morphology and mitochondrial content (number and protein levels)^31^. Several studies have further reported that alterations in mtDNA copy number (mtDNAcn), an essential precursor for biogenesis, occur in aging organs and in neurodegenerative disorders^31^. Increased accumulation of mtDNA mutations and damage have been shown to contribute to impaired mitochondrial function and aging^32,33^. At the single-cell level, OXPHOS failure becomes apparent when the pathogenic mtDNA mutations and deletions exceed a threshold level of 60–90%^34^. Cells may compensate for the age-related mitochondrial dysfunction by using dynamic compensatory and regulatory mechanisms such as mitochondrial biogenesis and mitophagy, which are processes critical for maintaining the functional and structural integrity of post-mitotic tissues such as the brain and skeletal muscle^34^.

Although studies on the brain are prioritized in HD, one must consider the importance of other peripheral organs. Muscle wasting is an important component of HD pathology and ultimately contributes to the demise of individuals with HD^35^. Studies on mice allow us the opportunity to make direct comparisons between the brain and skeletal muscle tissue and to assess the effects of both genotype and age on these tissues, which are affected in HD. In the present study, we used the YAC128 model of HD to examine the effects of mHTT on mitochondrial parameters in the brain and skeletal muscle. The YAC128 mouse expresses full-length human mHTT under endogenous regulatory control and recapitulates many symptoms of HD including motor and cognitive abnormalities with neuropathological features including selective striatal degeneration^36–38^. These mice display cognitive dysfunction at 2 months of age and develop motor abnormalities from 3 months of age with increased activity in the open field test, followed by increased motor impairment by 6 months of age. Progressive neuropathological changes occur with a measurable reduction in brain and striatum volume already from 3 months of age.

We hypothesized that affected tissues in HD are rendered vulnerable by altered mitochondrial dynamics. To test our hypothesis and to understand the natural changes that occur with aging and in the presence of mHTT, we assessed multiple mitochondrial parameters including morphological changes, mtDNAcn, mtDNA damage, mitochondrial function and transcript levels of genes involved in the mitochondrial dynamics in striatum, cortex and skeletal muscle of YAC128 and wild-type (WT) mice.

## Results

### Age-related increase in mitochondrial and lysosomal volume fractions in striatal neurons

**Fig. 1:**
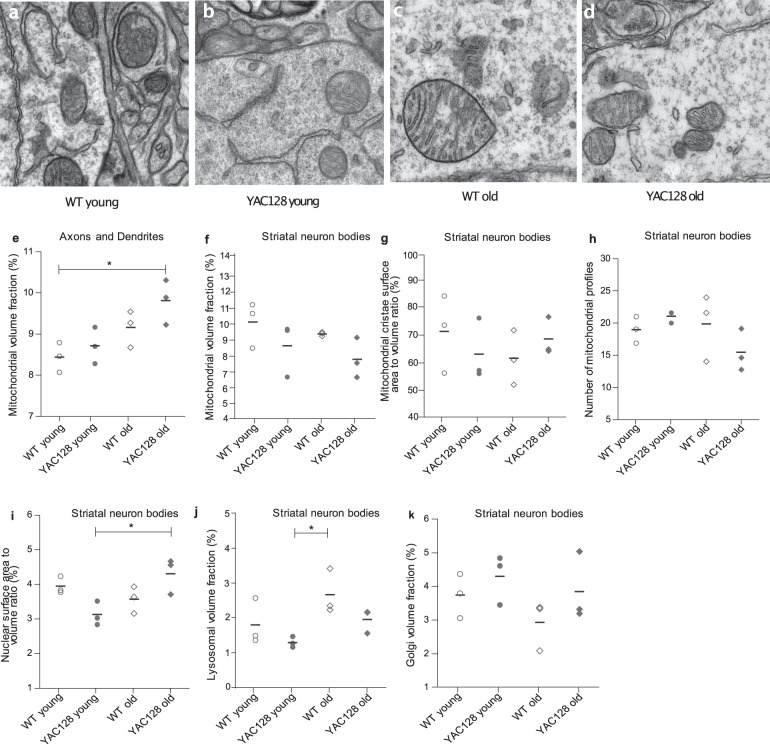
Ultrastructural assessment of quality of mitochondria and mitochondrial mass. Striatal tissue was analyzed using transmission electron microscopy (TEM). Images randomly selected of striatal cell body with mitochondria, nuclear envelope and ER at ×46,000 magnification from **a** young WT mouse, **b** young YAC128 mouse, **c** old WT mouse and **d** old YAC128 mouse. **e** Age had a significant effect on mitochondrial volume fraction (%) in the axon/dendrite regions adjacent to striatal neuron cell bodies. **f** Age and genotype had no effect on mitochondrial volume fractions of striatal neuron cell bodies, **g** mitochondrial cristae surface area to volume ratio of striatal neuron cell bodies, and **h** the number of mitochondrial profiles in striatal neuron cell bodies. **i** There was a significant age × genotype interaction effect on the nuclear surface area to volume ratio in striatal neuron cell bodies, which was increased in old YAC128 compared to young YAC128 mice. **j** Age had a significant effect on lysosomal volume fraction (%) in striatal neuron cell bodies. **k** Age and genotype had no effect on Golgi volume fractions in striatal neuron cell bodies. Dot plot shows dots where each dot represents the mean of 15 counted cell bodies or axon/dendrites. *n* = 3 mice per group. The horizontal line indicates the mean. Two-way ANOVA with Tukey’s multiple comparison test was used for analysis. *p < 0.05.

Mitochondrial biogenesis can be defined as the growth and division of pre-existing mitochondria and is accompanied not only by variations in number but also in mitochondrial size and mass^39^. We set out to test our hypothesis that mitochondrial biogenesis and function may be affected in YAC128 HD mice by first assessing mitochondrial content in striatal tissue, which selectively degenerates in HD, using transmission electron microscopy (TEM). We performed a qualitative analysis of the striatal tissue in young (1 month) and old (21–23 months) YAC128 and WT mice, which showed no obvious ultrastructural differences of mitochondria between any of the groups at magnifications between ×5800 and ×46,000 (Fig. 1a−d). Qualitatively there were no differences noted in mitochondrial morphological structures, for example, outer membrane, inner membrane, cristae, matrix, etc. between the groups. It should be noted that the purpose of the representative images is not to imply that the mitochondria differ in size. We quantified mitochondria and related organelles, that is lysosomes, Golgi and nuclei within cell bodies, by assessing the volume fractions using point counting. A two-way analysis of variance (ANOVA) was conducted, indicating an effect of age but not genotype, on the volume fraction of mitochondria in the axon/dendrite regions (age *F*_(1,8)_ = 12.0, *p* = 0.009; genotype *F*_(1,8)_ = 3.1, *p* = 0.117; interaction *F*_(1,8)_ = 0.50, *p* = 0.50) (Fig. 1e). However, there was no significant difference in mitochondrial volume fractions between old YAC128 (mean ± s.e.m; 9.81 ± 0.32%), young YAC128 (8.71 ± 0.26%) and old WT (9.16 ± 0.26%) mice. There were no effects of age or genotype on the volume fractions of mitochondria in the striatal cell body cytoplasm (age *F*_(1,8)_ = 1.17, *p* = 0.31; genotype *F*_(1,8)_ = 4.26, *p* = 0.073; interaction *F*_(1,8)_ = 0.01, *p* = 0.94) and no significant differences between the means of old YAC128 (7.70 ± 0.72%), young YAC128 (8.56 ± 0.98%) and old WT (9.28 ± 0.07%) mice (Fig. 1f). There were further no effects of age or genotype on the surface area to volume ratios of mitochondrial cristae (Fig. 1g) or the number of mitochondrial profiles (Fig. 1h) in striatal neuron cell bodies. The two-way ANOVA analysis revealed a significant age × genotype interaction effect, but did not identify any effects of age or genotype on the nuclear surface area to volume ratio of striatal neuron cell bodies (age *F*_(1,8)_ = 3.2, *p* = 0.11; genotype *F*_(1,8)_ = 0.03, *p* = 0.859; interaction *F*_(1,8)_ = 11.9, *p* = 0.009), whereby the nuclear surface area volume ratios increased with age, but only in the YAC128 mice, with mean volume ratios of 4.30 ± 0.30% in the old YAC128 and 3.11 ± 0.20% in the young YAC128 mice (Fig. 1i). The mean nuclear surface area to volume ratio was 3.93 ± 0.14% in young WT and 3.56 ± 0.22% in old WT mice.

Mitophagy is the selective autophagic mechanism inside lysosomes that eliminates damaged or redundant mitochondria, with lysosomes coming from the Golgi apparatus^40^. Altered Golgi volume fractions, if present, could indicate that they are pumping out more or fewer lysosomes. Lysosomal behavior may change once they are produced, for example, they can enlarge. We therefore measured the volume fractions of both these organelles in the cell bodies. The two-way ANOVA indicated an effect of age on lysosomal volume fractions in striatal neuron bodies, while the genotype effect was not significant (age *F*_(1,8)_ = 6.9, *p* = 0.031; genotype *F*_(1,8)_ = 4.4, *p* = 0.070; interaction *F*_(1,8)_ = 0.12, *p* = 0.74) (Fig. 1j). There was, however, no significant difference in mean lysosomal volume fraction between the old WT (2.67 ± 0.38%), the young WT (1.80 ± 0.39%) and the old YAC128 (1.96 ± 0.20%) mice. There was no effect of age or genotype on the volume fraction of the Golgi apparatus (Fig. 1k). In summary, we found no overt differences in mitochondrial morphological structures between the age- and genotype groups. Meanwhile, we found a positive correlation between aging and volume fraction of mitochondria located in axons/dendrite regions and lysosomes in striatal neuron cell bodies.

### Reduced mtDNA copy number in cortex and skeletal muscle of YAC128 mice

**Fig. 2:**
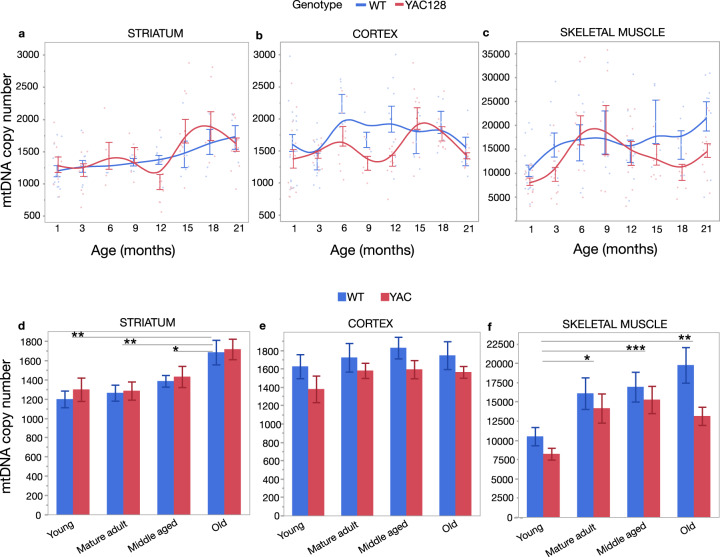
Quantification of mtDNA copy number (mtDNAcn) in striatum, cortex and skeletal muscle. For each DNA sample, the single-copy nuclear gene for the polymerase gamma 2 accessory subunit (*Polg2*) and the mitochondrial gene for cytochrome c oxidase 1 subunit gene (*mt-Co1*) were quantified by qPCR and presented as mtDNA:nDNA ratio. **a** In the striatum, the mtDNAcn increased in an age-dependent manner with no difference between the YAC128 and WT mice. **b** In the cortex, the mtDNAcn increased between 1 and 9 months of age followed by a decrease between 9 and 21 months of age, with significantly lower mtDNAcn in the YAC128 mice. **c** The mtDNAcn in skeletal muscle increased significantly between 1 and 9 months of age, followed by a decrease between 9 and 21 months of age, with significantly lower mtDNAcn in the YAC128 mice. Lines represent the fitted means of WT (blue) and YAC128 (red) with s.e.m. The sample size ranges from 4 to 18 per group at each time point (for striatum; WT 6 months, *n* = 1) (see also Supplementary Tables 1–3). **d** In the striatum, age had a significant effect on mtDNAcn with no effect of genotype. The mtDNAcn was significantly increased in old mice (18–21 months) compared to young (1 month), mature adult (3–6 months) and middle-aged mice (9–15 months). **e** In the cortex, genotype had a significant effect with no effect of age with the YAC128 mice displaying significantly lower mtDNAcn compared to the WT mice. **f** In the skeletal muscle, both age and genotype had a significant effect on mtDNAcn. There was a progressive increase in mtDNAcn in mature adult, middle-aged and old mice compared to young mice, while the mtDNAcn was significantly lower in the YAC128. Multivariate regression analysis, fitting age as a categorical variable, with Tukey’s multiple comparison test was used for analysis. Bars represent means with s.e.m. WT (blue) and YAC128 (red). ***p < 0.001, **p < 0.01, *p < 0.05.

Alterations in mtDNAcn are thought to reflect mitochondrial biogenesis and increases may occur in response to impairments in mitochondrial function^31^. To investigate further whether a mitochondrial biogenesis mechanism is occurring, we used a PCR-based approach to assess mtDNA content allowing us to analyze larger sample sizes and additional ages. We next analyzed mice spanning the age range of 1−21 months of age, in 3-month increments. Overall, the mtDNAcn increased with age in striatum (*t*_(122)_ = 4.52, *p* < 0.0001) with no significant effect of genotype (Fig. 2a) (Table 1). Subanalysis of the genotype groups showed that striatal mtDNAcn is a linear function of age in WT, while YAC128 displayed a non-linear function with significant changes in mtDNAcn between 12 and 21 months of age (Supplementary Table 1). In cortex, the mtDNAcn increased up to 9 months of age (*t*_(142)_ = 2.71, *p* = 0.008), followed by a decrease to 21 months of age (*t*_(142)_ = −2.23, *p* = 0.027) (Table 1) (Fig. 2b). There was a genotype effect with significantly lower mtDNAcn in YAC128 mice compared to WT (*t*_(142_) = −2.55, *p* = 0.012) (Table 1). The mtDNAcn was lower in YAC128 at 6, 9 and 12 months of age when time points were analyzed individually (Supplementary Table 2). Subgroup analysis showed that the mtDNAcn significantly increased up to 6 months of age in WT mice (*t*_(60)_ = 2.13, *p* = 0.037) and leveled thereafter, while there were significant fluctuations in mtDNAcn across the full age range in the YAC128 mice (Supplementary Table 1).

**Table 1:** Effects of age and genotype on mtDNA copy number in striatum, cortex and skeletal muscle of YAC128 and WT mice.

Tissue	Variables	Spline^a^	Coef.	SE	*t* value	*p* value	95% CI^b^
Striatum^c^	Age		23.7	5.25	4.52	<0.0001	13.3–34.1
	Genotype		38.3	74.2	0.52	0.607	−108.7–185.3
Cortex^d^	Age spline 1	1–9	45.1	16.7	2.71	0.008	12.2–78.1
	Age spline 2	9–21	−59.2	26.6	−2.23	0.027	−111.7 to −6.72
	Genotype		−205.3	80.6	−2.55	0.012	−364.6 to −45.9
Skeletal muscle^d^	Age spline 1	1–9	864.1	263.8	3.28	0.001	342.5 – 1386
	Age spline 2	9–21	−929.5	405.9	−2.29	0.024	−1732 to −126.8
	Genotype		−2914	1249	−2.33	0.021	−5383 to −443.9

^a^Spline indicates the knots in the cubic spline regression.

^b^95% confidence interval.

^c^Linear regression analysis was used for the analysis of mtDNA copy number in the striatum (d.f. = 122).

^d^Cubic spline regression was used for the analysis of mtDNA copy number in the cortex (d.f. = 142) and skeletal muscle (d.f. = 143).

In skeletal muscle, mtDNAcn increased up to 9 months of age (*t*_(143)_ = 3.28, *p* = 0.001), followed by a significant decrease between 9 and 21 months of age (*t*_(143)_ = −2.29, *p* = 0.024) (Table 1) (Fig. 2c). There was a genotype effect with significantly lower mtDNAcn in skeletal muscle of YAC128 mice compared to WT (*t*_(143)_ = −2.33, *p* = 0.021) (Table 1). Notably, the YAC128 mice had lower mtDNAcn in skeletal muscle already at 3 months of age compared to WT (*t*_(18)_ = −2.20, *p* = 0.041) (Supplementary Table 2). Subgroup analysis showed that the mtDNAcn in WT increased with age in a linear manner (*t*_(64)_ = 3.21, *p* = 0.002). In contrast, YAC128 mice displayed significant fluctuations with increased mtDNAcn between 1 and 9 months (*t*_(78)_ = 3.11, *p* = 0.003), followed by a significant decrease between 9 and 21 months of age (*t*_(78)_ = −2.76, *p* = 0.007) (Supplementary Table 1). The average mtDNAcn was approximately 10- to 20-fold higher in the skeletal muscle compared to striatum and cortex (Supplementary Table 3).

The YAC128 mice display a mild cognitive impairment at 2 months of age, after which the disease progresses and the mice begin to exhibit behavioral and neuropathological changes similar to patients with HD^36,38,41^. We next categorized the mice into four different bins based on aging and disease severity to further determine the effects of age and genotype on mtDNAcn^42^. Young (1 month) YAC128 mice are asymptomatic, while mature adult YAC128 mice (3–6 months) display cognitive and motor dysfunction with neuropathological changes progressing in middle-aged (9–15 months) and old (18–21 months) transgenic mice. Multivariate regression analysis showed a significant effect of age but not genotype on mtDNAcn in striatum (age *F*_(3,117)_ = 6.51, *p* = 0.0004; genotype *F*_(1,117)_ = 0.44, *p* = 0.51) with significant differences between young (mean 1256 ± 87 copies; *t*_(117)_ = −3.73, *p* = 0.0017), mature adult (1272 ± 80 copies; *t*_(117)_ = −3.83, *p* = 0.0012) and middle-aged mice (1411 ± 60 copies; *t*_(117)_ = −2.90, *p* = 0.023) compared to old mice (1695 ± 78 copies) (Fig. 2d). In cortex, genotype had a significant effect on mtDNAcn with no effect of age between the groups (WT mean 1736 ± 63 copies vs. YAC128 mean 1530 ± 54 copies; age *F*_(3,137)_ = 1.19, *p* = 0.313; genotype *F*_(1,137)_ = 6.24, *p* = 0.0136) (Fig. 2e).

In skeletal muscle, age and genotype both had significant effects (age *F*_(3,138)_ = 6.41, *p* = 0.0004; genotype *F*_(1,138)_ = 5.91, *p* = 0.0163), with mtDNAcn progressively increasing in mature adult (mean 15,198 ± 1235 copies; *t*_(138)_ = −3.24, *p* = 0.0080), middle-aged (mean 16,153 ± 1130 copies; *t*_(138)_ = −3.93, *p* = 0.0008) and old mice (16,142 ± 1285 copies; *t*_(138)_ = −3.68, *p* = 0.0019) compared to young mice (mean 9306 ± 1329 copies) (Fig. 2f). In addition, the mtDNAcn was significantly lower in the YAC128 (mean 12,686 ± 839 copies) compared to WT mice (mean 15,714 ± 920 copies). From this analysis, we concluded that the mtDNAcn changes significantly with age and genotype in a tissue-specific manner. We found a positive correlation between aging and the mtDNAcn in striatum and skeletal muscle. Notably, the YAC128 mice had lower mtDNAcn in cortex and skeletal muscle.

### mtDNA deletions detectable in young and old YAC128 and WT mice

**Fig. 3:**
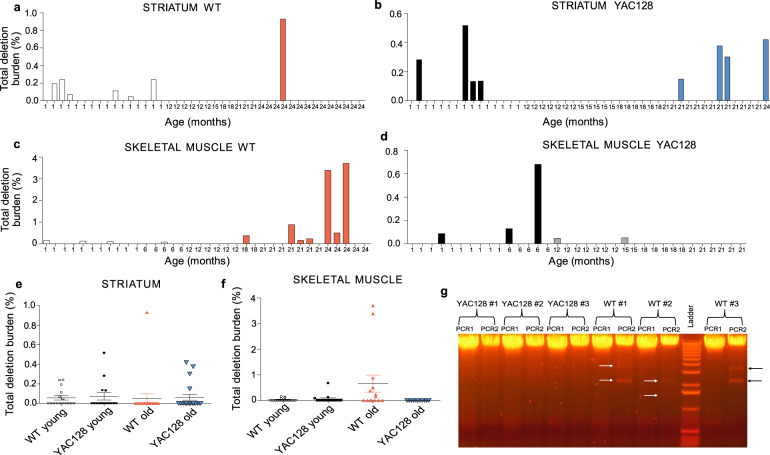
Distribution of fractions of deleted mitochondrial DNA (Δ-mtDNA) in striatum and skeletal muscle tissue. **a**−**d** Each bar represents the burden of Δ-mtDNA as measured by long-range PCR at the indicated age. Zero-height bars represent samples that were determined to be deletion-free by long-range PCR. In total, **a** 6/16 WT and **b** 4/15 YAC128 mice had Δ-mtDNA in striatum at 1 month of age, while **c** 3/11 WT mice and **d** 1/10 YAC128 mice had Δ-mtDNA in skeletal muscle. **e** Age had an effect with increased odds for Δ-mtDNA in striatum of young mice (odds = 3.24, *p* = 0.048). **f** Genotype had an effect with increased odds for Δ-mtDNA in skeletal muscle of WT mice (odds = 4.44, *p* = 0.026). **g** Representative image of Δ-mtDNA bands in skeletal muscle of three old YAC128 mice (YAC128 #1–3) and three old WT mice (WT #1–3) (21–24 months of age). For each DNA sample, the left lane represents PCR product 1 (PCR1) and the right lane represents PCR product 2 (PCR2). Arrows in black and white indicate detected bands. Number of mice analyzed in striatal tissue: WT young (1 month) *n* = 16; YAC128 young (1 month) *n* = 15; WT old (18–24 m) *n* = 19; YAC128 old (18–24 months) *n* = 20; Skeletal muscle (quadriceps): WT (1 month) *n* = 11; YAC128 (1 month) *n* = 10; WT (6 months) *n* = 5; YAC128 (6 months) *n* = 5; WT old (18–24 months) *n* = 14; YAC128 old (18–21 months) *n* = 11. Binomial generalized linear models were applied for analysis.

We next assessed the mtDNA damage by quantifying the total burden of mtDNA molecules with deletions. We detected deleted mtDNA (Δ-mtDNA) in striatum (Fig. 3a, b) in both WT and YAC128 mice already at 1 month of age. In total, 6/16 WT and 4/15 YAC128 mice had Δ-mtDNA in striatum. Age, but not genotype had an effect, with a significantly increased probability for Δ-mtDNA in striatum of young mice (odds = 3.24, *p* = 0.048) (Fig. 3e). In skeletal muscle, 3/11 WT mice and 1/10 YAC128 mice had Δ-mtDNA at 1 month of age (Fig. 3c, d). Genotype, but not age had an effect, with an increased probability for Δ-mtDNA in WT mice (odds = 4.44, *p* = 0.026) (Fig. 3f). Notably, although we observed Δ-mtDNA bands in skeletal muscle of aged WT mice (Fig. 3g), none of the old YAC128 mice had any detectable Δ-mtDNA. Thus, although Δ-mtDNA was detected in both young and old mice, there was a higher probability to detect striatal Δ-mtDNA in young mice, while this was not observed in skeletal muscle.

### Age-related accumulation of COX-deficient muscle fibers in YAC128 and WT mice

**Fig. 4:**
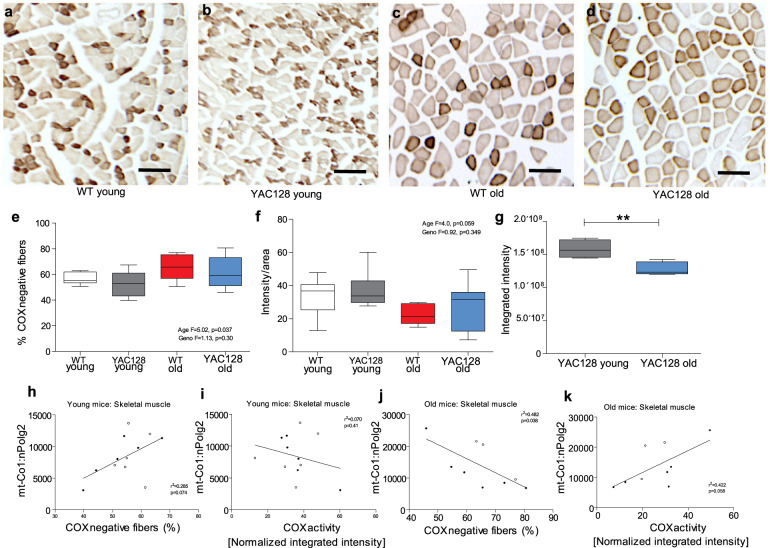
Age-related decrease in cytochrome c oxidase (COX) deficiency in striatum and skeletal muscle. Cytochrome c oxidase staining was performed on frozen sections of quadriceps from **a** young WT (1 month), **b** young YAC128 (1 month), **c** old WT (21 months) and **d** old YAC128 (21 months) (representative images). Scale bar = 250 μm. **e** The numbers of COX-negative fibers increased in an age-dependent manner. **f** Semi-quantitative assessment of staining intensity showed a borderline significant age-related decrease in aged mice. **g** In striatal tissue, the COX-staining intensity was decreased in aged YAC128 (*n* = 5) compared to young YAC128 mice (*n* = 4). **h** Correlation between the mtDNAcn and the number of COX-negative muscle fibers in young WT (*n* = 6) and YAC128 mice (*n* = 6). **i** Correlation between the mtDNAcn and COX-staining intensity in young WT (*n* = 6) and YAC128 mice (*n* = 6). **j** Correlation between the mtDNAcn and the number of COX-negative muscle fibers in old WT (*n* = 3) and YAC128 mice (*n* = 6). **k** Correlation between the mtDNAcn and COX-staining intensity in old WT (*n* = 3) and YAC128 mice (*n* = 6). Young mice = 1 month; old mice = 18–22 months of age. Open circles represent WT; closed circles represent YAC128. Two-way ANOVA with Tukey’s multiple comparison test, unpaired two-sided *t* test and simple linear regression were used for statistical analysis. **p < 0.01.

Cytochrome c oxidase (COX) is a partially mtDNA-encoded enzymatic complex central to oxidative phosphorylation. To study the functionality of the mitochondrial respiration efficiency, we next measured COX activity in young (1 month) and old (18–22 months) YAC128 and WT mice in skeletal muscle (Fig. 4a−d). There was an age-related increase in the number of COX-negative skeletal muscle fibers, but with no genotype effect (age *F*_(1,20)_ = 5.02, *p* = 0.037; genotype *F*_(1,20)_ = 1.13, *p* = 0.30) (Fig. 4e). We found similar tendencies when measuring the COX histochemical staining intensity in skeletal muscle tissue sections (age *F*_(1,20)_ = 4.0, *p* = 0.059; genotype *F*_(1,20)_ = 0.92, *p* = 0.35) (Fig. 4f). In striatal tissue, the COX-staining intensity was decreased in old compared to young YAC128 mice (*p* = 0.0073) (Fig. 4g). The striatum was not quantified as positive and/or negative neurons as the density of the MSNs in the striatal tissue did not allow for this classification using immunohistochemistry. These data suggested that COX-deficient cells increased in an age-related manner independent of genotype, at least in skeletal muscle fibers.

We next investigated the relationship between COX-staining intensity and mtDNA content. We analyzed young and old mice separately, by combining WT and YAC128 mice in these two age categories due to the lack of obvious genotype effects. In old mice, we observed a moderate negative correlation between the mtDNAcn and the number of COX-negative skeletal muscle fibers (*r*^2^ = 0.48) (Fig. 4j) and a moderate positive correlation between the mtDNAcn and COX-staining intensity (*r*^2^ = 0.42) (Fig. 4k). In young mice these relationships were weak or absent (Fig. 4h, i). These results show a mitochondrial biochemical defect at the cellular level readily observed as an increased number of COX-negative fibers in skeletal muscle and as reduced COX-intensity in skeletal muscle and striatum of old mice independently of genotype, at least in skeletal muscle fibers.

### ATP and ADP levels are unaffected in YAC128 mice

**Fig. 5:**
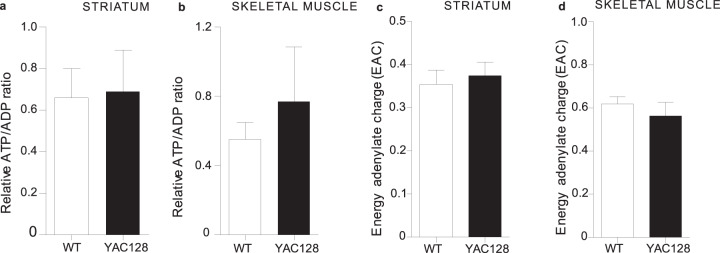
ATP production is unaltered in the YAC128 mice compared to WT mice at 12 months of age. Genotype has no effect on the ATP synthase activity as measured by relative ATP/ADP ratio values in **a** striatum and **b** skeletal muscle using etheno derivatization/HPLC or on the levels of total mass of adenylate nucleotides (TAN) in **c** striatum or **d** skeletal muscle. Data are presented as mean ± s.e.m. WT *n* = 6; YAC128 *n* = 9. Unpaired two-sided *t* test was used for statistical analysis.

Adenine nucleotides are major determinants of the energy status of the cell and thus any modulation of their cellular concentration may impact cellular metabolism and growth. As ATP synthesis is dependent on mitochondrial function, we next measured adenylate nucleotide levels in YAC128 and WT mice at 12 months of age, an age at which YAC128 mice exhibit both behavioral and neuropathological deficits^36^. The relative ATP/ADP ratios, an indicator of cellular energy status and mitochondrial bioenergetic function, were comparable in YAC128 and WT mice in striatum (Fig. 5a) and skeletal muscle (Fig. 5b). We also quantified the adenylate energy charge (AEC), a parameter describing the energy status in cells and tissues and defined as [[ATP + (0.5ADP)]/(AMP + ADP + ATP)]^43^. We observed comparable AEC levels in YAC128 and WT mice at 12 months of age in both striatum (Fig. 5c) and skeletal muscle tissue (Fig. 5d), indicating that the energetic status, synthesis and total mass of adenine nucleotides are unchanged in these tissues. These results are consistent with reports showing no difference in ATP or ADP content in brain mitochondria isolated from adult YAC128 mice^44^, although a difference in ATP production at later stages cannot be excluded.

### Age and genotype-related expression changes in mitochondria-related genes

**Fig. 6:**
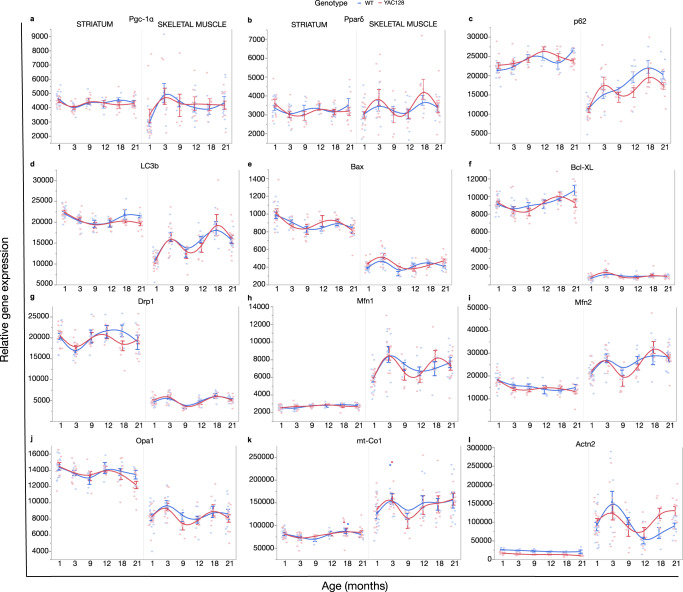
Age and genotype effects on transcript levels of genes involved in mitochondrial dynamics. Lines represent the fitted means of WT (blue) and YAC128 (red) with s.e.m. **a** Age and genotype had no effect on *Pgc-1α* levels in striatum. Age-related changes on *Pgc-1α* transcript levels with no genotype effect in skeletal muscle. **b** Age and genotype had no effect on *Pparδ* transcript levels in striatum and skeletal muscle. **c** Age-related changes in *p62* transcript levels with no genotype effect in striatum and skeletal muscle. **d** Age-related changes in *LC3b* transcript levels with no genotype effect in striatum and skeletal muscle. **e** Age-related changes in *Bax* transcript levels with no genotype effect in striatum. Age- and genotype-related changes in *Bax* transcript levels in skeletal muscle. **f** Age-related changes in *Bcl-xL* transcript levels with no genotype effect in striatum. Age and genotype had no effect on *Bcl-xL* expression in skeletal muscle. **g** Age-related changes in *Drp1* transcript levels with no genotype effect in striatum and skeletal muscle. **h** Age-related changes in *Mfn1* expression with no genotype effect in striatum and skeletal muscle. **i** Age-related changes in *Mfn2* expression with no genotype effect in striatum and skeletal muscle. **j** Age-related changes in *Opa1* transcript levels with no genotype effect in striatum and skeletal muscle. **k** Age and genotype had no effect on *mt-Co1* transcript levels in striatum. Age-related changes in *mt-Co1* transcript levels in skeletal muscle. **l** Age- and genotype-related changes in *Actn2* transcript levels in striatum. Age-related changes in *Actn2* transcript levels in skeletal muscle. Sample size ranges from n = 3–16. See also Supplementary Tables 4–6.

We next analyzed genes associated with apoptosis, autophagy, mitochondrial biogenesis, fusion, fission and MRC, by measuring transcript levels in striatum and skeletal muscle tissue of YAC128 and WT mice at 1–21 months of age. *Pgc-1α* is a transcriptional co-activator and a key regulator of mitochondrial biogenesis, oxidative phosphorylation and fiber-type switching in skeletal muscle^45,46^, shown to be dysregulated in human HD and other HD models^47,48^. There was no effect of age or genotype on the mRNA levels of *Pgc-1α* in striatum (Fig. 6a) (Supplementary Table 4). In skeletal muscle, we found age-dependent changes in *Pgc-1α* mRNA levels significantly increasing up to 3 months (*p* = 0.024), while decreasing between 3 and 21 months of age (*p* = 0.046) in YAC128 and WT mice. There was no effect of age or genotype on *Pparδ* mRNA levels in striatum or skeletal muscle (Fig. 6b) (Supplementary Table 4), a gene suggested to also regulate mitochondrial biogenesis and oxidative capacity^49^. We measured *p62* and *LC3b*, which are markers for autophagic activity^50^. The autophagy receptor *p62* has the ability to bind to ubiquitinated proteins and *LC3b*, which is recruited to the autophagosomal membranes. Striatal p62 mRNA levels increased significantly between 1 and 12 months of age (*p* < 0.0001) followed by a decrease between 12 and 21 months of age (*p* = 0.004), with no effect of genotype. In skeletal muscle, the *p62* mRNA levels changed significantly with age in a non-linear fashion with no effect of genotype (Fig. 6c) (Supplementary Table 4). YAC128 mice expressed significantly lower p62 mRNA levels in striatum (*p* = 0.0053) and skeletal muscle (*p* = 0.046) at 21 months of age (Supplementary Table 5). *LC3b* also showed age-related transcriptional changes but with decreased striatal levels between 1 and 9 months of age (*p* < 0.0001), followed by increased levels between 9 and 21 months (*p* = 0.001), without evidence for genotype effects (Fig. 6d) (Supplementary Table 4). In skeletal muscle, the *LC3b* mRNA levels increased between 1 and 3 months of age (*p* < 0.0001), followed by a decrease between 3 and 9 months of age (*p* < 0.0001) and then increased levels again between 9 and 21 months of age (*p* < 0.0001). Expression levels of *Bax*, a transcript encoding a protein that promotes pro-apoptotic actions while interacting with mitochondria, showed significant age-dependent changes in striatum (*p* < 0.0001) with no effect of genotype (Fig. 6e) (Supplementary Table 4). In skeletal muscle, the *Bax* mRNA levels increased between 1 and 3 months of age (*p* < 0.0001), decreased between 3 and 9 months of age (*p* < 0.0001), and then increased between 9 and 21 months of age (*p* < 0.0001) with a significant genotype effect (*p* = 0.031). Interestingly, the YAC128 mice displayed significantly higher *Bax* transcript levels in skeletal muscle already at 1 month of age (*p* = 0.012) (Supplementary Table 5). For *Bcl-XL*, which has anti-apoptotic properties by regulating the release of cytochrome c from the mitochondria, the transcript levels fluctuated and increased with age in striatum (*p* = 0.011) with no effect of genotype (Fig. 6f) (Supplementary Table 4). There were no age- or genotype-dependent changes in *Bcl-XL* transcript levels in skeletal muscle. Large GTPases of the dynamin family mediate mitochondrial fission and fusion; *Drp1* mediates mitochondrial fission, while *Opa1* and the mitofusins, *Mfn1* and *Mfn2*, mediate mitochondrial fusion. *Drp1* showed fluctuating age-related expression changes in striatum with decreased levels between 1 and 3 months (*p* = 0.003), followed by increased levels between 3 and 12 months of age (*p* = 0.001) and again decreased levels between 12 and 21 months of age (*p* = 0.001) (Fig. 6g) (Supplementary Table 4). In skeletal muscle, *Drp1* mRNA levels fluctuated significantly increasing between 1 and 3 months of age (*p* < 0.0001), decreasing between 3 and 9 months of age (*p* < 0.0001), followed by an increase between 9 and 18 months of age (*p* < 0.0001) and decreasing again at 18–21 months of age (*p* < 0.0001). *Mfn1* striatal transcript levels increased significantly with age (*p* = 0.010) with no effect of genotype (Fig. 6h) (Supplementary Table 4). There were significant age effects on *Mfn1* expression in skeletal muscle with increased mRNA levels between 1 and 3 months (*p* < 0.0001) that decreased between 3 and 12 months of age (*p* < 0.0001) and then increased again between 12 and 21 months of age (*p* < 0.0001). *Mfn2* mRNA levels in striatum decreased significantly with age (*p* < 0.0001). In skeletal muscle, *Mfn2* mRNA levels fluctuated with age with increased levels between 1 and 3 months of age (*p* = 0.015), decreased levels between 3 and 9 months of age (*p* = 0.0024) and increased levels between 9 and 21 months of age (*p* = 0.0024) (Fig. 6i) (Supplementary Table 4). *Opa1* striatal mRNA expression levels changed with age with decreased levels between 1 and 9 months of age (*p* = 0.002), increased between 9 and 12 months of age (*p* = 0.002) and decreased levels between 12 and 21 months of age (*p* = 0.002) with no effect of genotype (Fig. 6j) (Supplementary Table 4). The *Opa1* mRNA levels in skeletal muscle increased between 1 and 3 months of age (*p* = 0.008), decreased between 3 and 9 months of age (*p* = 0.003) and increased between 9 and 21 months of age (*p* = 0.003) with no genotype effect. *mt-Co1*, that is a component of the MRC with the potential to cause mitochondrial complex IV deficiency as it is one of three subunits in complex IV, showed no age- or genotype-related expression changes overall in striatum (Fig. 6k). Striatal *mt-Co1* mRNA levels were, however, significantly higher in YAC128 at 9 months of age (Supplementary Table 5). In skeletal muscle, *mt-Co1* increased significantly between 1 and 3 months of age (*p* = 0.043), decreased between 3 and 9 months of age (*p* = 0.042) and increased between 9 and 21 months of age (*p* = 0.042). Provided the involvement of actin microfilaments in mitochondrial transport and our previous observation that *Actn2* is dysregulated in YAC mouse striatum^51^, we here attempted to confirm our previous finding by additionally measuring the expression levels in skeletal muscle. In striatum, the *Actn2* mRNA levels decreased significantly with age (*p* < 0.0001) (Fig. 6l). In addition, YAC128 mice showed significantly lower expression levels compared to WT mice at every time point analyzed individually (Supplementary Table 5). In skeletal muscle, *Actn2* transcriptional changes were also age-related, but with no overall genotype effect. The *Actn2* mRNA levels increased significantly between 1 and 3 months of age (*p* = 0.010), decreased between 3 and 12 months of age (*p* = 0.001) and increased between 12 and 21 months of age (*p* = 0.001). At variance with striatum, YAC128 mice expressed significantly higher *Actn2* mRNA levels compared to WT mice at 18 and 21 months of age when time points were analyzed individually. From this analysis, we concluded that there were no overt genotype-related expression differences in the fission and fusion regulatory genes or *Pgc-1α*, a possible regulator of mtDNAcn, that would explain the reduced mtDNAcn in YAC128 skeletal muscle.

## Discussion

This study demonstrates that the mitochondrial dynamics are altered in aging YAC128 mice. Morphometric analysis, although limited by a small sample size, suggested increased volume fractions of mitochondria in the striatal neurites of old mice with an age-related increase in lysosomal volume fraction. Together, the data presented might suggest that the motility of the mitochondria is reduced, that mitochondrial transport is impaired or that the potential for clearance through mitophagy decreases with age in MSNs. Previous studies have indeed shown that the wild-type HTT has the ability to promote both antero- and retrograde mitochondrial transport, while mHTT disrupts the formation of transport complexes and impairs mitochondrial movement and transport contributing to HD pathology^6,7,17,52^. However, recent studies by Van Laar et al.^53^ have reported data supporting the concept of compartmentalized axonal mitochondrial biogenesis. The authors provide compelling evidence for mitochondrial biogenesis occurring not only in the cell bodies, with new mitochondria transported down axons as generally presumed, but also in the distal axons^53,54^. They demonstrated that rotenone exposure of primary cortical rat neurons upregulates mtDNA replication as an early response to neurodegeneration-relevant stress, specifically in distal axonal regions where neurodegeneration starts. Interestingly, we found an age-related increase in mitochondrial volume fractions in the axon/dendrite regions of old mice. Thus, this could potentially reflect compartmentalized axonal mitochondrial biogenesis occurring as described by Van Laar’s et al., although this should be considered purely hypothetical and further investigations on a larger confirmatory study are required to test this hypothesis.

Alterations in the mtDNAcn have been associated with aging and neurodegenerative disorders^31^. Consequently, the mtDNA content might represent a useful early biomarker to monitor changes in different physiological and pathological states. We here show that the mtDNAcn changes significantly with age and genotype in a tissue-specific manner. We found that striatal mtDNAcn was significantly increased in old WT and YAC128 mice. Notably, the increased mtDNAcn in old mice corresponded to increased mitochondrial volume fraction in axons/dendrites regions adjacent to the striatal neuron cell bodies, suggestive of mitochondrial biogenesis occurring at a later age in striatum. Of note is that we did not find any age- or genotype-related changes in mRNA levels of *Pgc-1a* in striatum, which is a key player in mitochondrial biogenesis that has been shown to be transcriptionally altered in other HD models and caudate MSNs^48^. Rice et al.^55^ showed that the relationship between expression levels of mitobiogenesis signaling factors appeared to be intact in hippocampal pyramidal neurons from control subjects, whereas this relationship is disrupted in AD cases. Hence, additional factors including *Nfr1, Nfr2*, *Tfam* and *ERRα* will be examined in future studies to determine if these contribute to the age-related increase in striatal mtDNAcn. Remarkably, the cortical mtDNAcn was significantly lower in YAC128 compared to WT. In contrast to our findings, Hering et al.^56^ reported lower mtDNAcn in the striatum with no difference in cortex of R6/2 HD. The authors also showed a higher relative mtDNAcn in the striatum compared with cortex^56^, which we did not observe in our study. The cell-type composition of a tissue can change due to pathology or aging^31^. Cell-type-specific differences due to neuronal loss and reactive gliosis may thus explain the differences observed between brain tissues and other HD models. Previous studies have shown that GFAP staining, which detects astrocytes and measures reactive astrogliosis, increased with age in striatum in both YAC128 and WT, while GFAP expression was more intense in the cortex of the YAC128 ^57^. The authors suggest that the reactive astrogliosis in cortex may be the outcome of the cell loss in striatum and not related to the mHTT. Interestingly, Rice et al.^55^ showed mtDNAcn changes in different cell types with significantly decreased mtDNAcn in hippocampal pyramidal neurons from AD cases, with a tendency towards reduced mtDNAcn GFAP + glia. Further investigation including cell-type-specific analysis is thus necessary to elucidate the discordance between brain regions and a possible link between reactive astrogliosis and the reduced mtDNAcn in the cortex of YAC128.

Cell-type-specific variation in mtDNAcn might moreover have pathological consequences. Microglial activation in the brain is a hallmark of HD and studies have shown increased microglial activity with altered morphology in the striatum of YAC128 at 12 months of age^58,59^. Castellani et al.^60^ emphasize the link between immune dysfunction, inflammation, energy demands and mitochondrial dysfunction and the potential effect reduced mtDNAcn might have on macrophage polarization and its consequences. M1 macrophages generate ATP through glycolysis while M2 macrophages use oxidative phosphorylation. Reduced mtDNAcn may lead to mitochondrial dysfunction with an inadequate number of mtDNA copies to encode for proteins of the cellular respiration. As a result, macrophages may lose their ability to switch to an anti-inflammatory M2 subtype, leading to a reduction in anti-inflammatory cytokine mediators that would assist in dampening of inflammation and tissue repair.

On the other hand, discrepancies might also be due to the genetic composition of cell and animal HD models with full-length or truncated mHTT combined with different expression levels that might influence mitochondrial parameters distinctively. Different methodological approaches and quantification methods may also have profound effects on the outcome measurements^31,61^. Consequently, further investigation of HD models and cell lines derived from HD patients is necessary to elucidate the underlying causes for biological and genetic discrepancies.

In skeletal muscle, we found a progressive age-related increase in mtDNAcn independent of genotype. However, mtDNAcn was significantly lower in old YAC128 compared to old WT mice. As mitochondrial biogenesis may represent an attempt by cells to increase their aerobic set point or to maintain a pre-existing aerobic set point in the face of declining mitochondrial function^34^, the overall increased mtDNAcn in the cortex and skeletal muscle observed in WT might suggest that compensatory biogenesis is occurring in the WT, but is not occurring to the same degree in the YAC128 mice in these tissues. However, the increased levels of mtDNAcn seen with age in skeletal muscle could alternatively be due to a muscle fiber switch, that is from fast glycolytic (type II) to slow oxidative mitochondria-rich fibers (type I)^62^. Skeletal muscle display phenotypic plasticity with the ability to change in response to the physiological and pathological changes, for example, studies on skeletal muscle diseases have shown switches in muscle fiber type, which may represent a compensatory mechanism to counteract disease progression and serve as a protective mechanism^62^. In HD, studies have shown a significant reduction in oxidative type I fibers and an increase in glycolytic type IIB fibers in HD mice, suggesting that mHTT hinders a muscle fiber switch^63^. One noteworthy observation in our study is the lack of difference in ATP/ADP ratio at 12 months of age and in the number of COX-negative muscle fibers and COX-intensity, despite significantly lower mtDNAcn in YAC128 muscle. This contrasts with previous findings showing reduced maximum rate of ATP production in skeletal muscle of HD patients^23^. The reason for this discrepancy is not clear, but might be related to fiber-type-specific differences or methodological differences as Lodi et al.^23^ used magnetic resonance spectroscopy on calf tissue. Nevertheless, our findings suggest that the energy production load is greater in YAC128 muscle since lower mtDNAcn would suggest less mitochondrial mass. This could potentially lead to greater oxidative stress in HD muscle resulting in muscle wasting and an HD muscle phenotype resembling a prematurely aged muscle^64^. Part of elucidating the underlying mechanisms and the consequences of the reduced mtDNAcn in skeletal muscle of YAC128 will entail assessments of additional regulatory factors. Of note is that we did not find any difference in transcript levels of *Pgc-1a*, suggesting that other factors might regulate the observed changes in mtDNAcn in YAC128 muscle. Supporting evidence for this comes from studies that assessed the expression levels of regulatory factors in different muscle fiber types in HD mice^63^. In gastrocnemius, a mixed fiber-type muscle comparable with quadriceps which we studied, *Tfam* transcript levels were significantly decreased while there was no difference in *Pgc-1a*, *Pgc-1β* and *Nrf1* transcript levels in HD mice. Hence, future studies will examine protein levels of these factors in a new cohort to ascertain if mitochondrial biogenesis is occurring in the skeletal muscle of YAC128 mice.

In contrast to compensatory activation of biogenesis, mitochondrial dysfunction may instead lead to cell death through apoptosis or increased autophagy of mitochondria, that is mitophagy^34^. For instance, morphometric analysis showed that the nuclear surface area to volume ratio is increased in old YAC128 but not in old WT, compared to young YAC128 mice. This finding might indicate that the nuclei volume decreases as the cells progress towards apoptosis involving the processes of chromatin clumping, loss of nucleoplasm and fragmentation of the nucleus in the old YAC128. In HD, autophagy failure may result in increased levels of pathogenic mHTT aggregations and reduced clearance of dysfunctional mitochondria^65,66^. To investigate whether autophagy is altered in the YAC128 mice, we measured the *p62* transcript levels, which in conjunction with *LC3b* may be used as markers for autophagic activity. The p62 protein is one of the selective macro-autophagy receptors that binds to autophagosomes via interaction with LC3b and plays an essential role in the selective turnover of aggregated proteins and mitochondria^67^. Previous studies showed no difference in p62 protein levels in YAC128 at 12 months of age^68^. We report reduced *p62* mRNA levels in both striatum and skeletal muscle of YAC128 at 21 months of age, indicating transcriptional repression of *p62* at a later stage. It is important to note that transcript levels might not reflect the cellular protein levels^50,69^. These data, in conjunction with our morphometric analysis suggesting age-related increases in lysosomal volume fractions in striatal neuron bodies and in mitochondrial volume fractions in neurites of old mice, will require larger future studies to investigate if the clearance of mitochondria through mitophagy is altered in old YAC128 mice. Alternatively, p62’s role here could be apoptosis-related as this protein is also known to play a role in apoptosis^70^. Regardless, this model presents a number of queries that need to be resolved and examined in parallel with the theory of compartmentalized axonal mitochondrial biogenesis occurring.

Mitochondrial dynamics are dependent on several mechanisms including cytoskeletal interactions with microtubules and actin microfilaments for mitochondrial motility and the regulation of the balance between fission/fusion. Perturbations in mechanisms regulating these functions are involved in neurodegenerative diseases^71^. Here we replicated our previous work^51^ showing that *Actn2* transcript levels, which encodes a cross-linker of actin filaments with implications in actin organization, is significantly reduced in the striatum of YAC128 mice as early as 1 month of age and consistently across all ages. Interestingly, studies in rat showed that ACTN2 is linked to *N*-methyl-d-aspartate (NMDA) receptors and the actin cytoskeleton in striatum^72^, whereas *Actn2* knockdown inhibits post-synaptic density 95 (PSD-95) protein assembly in spines and leads to failure to respond to stimulation of the NMDA receptor^73^. One hypothesis would be that the reduced *Actn2* levels might alter the coupling of the NMDA receptors to the cytoskeleton with consequences specific to the striatopallidal system. Yet another hypothesis would be that the reduced *Actn2* in the presence of mHTT might contribute to altered mitochondrial transport based on its anchoring role in the actin microfilaments already at an early age in the striatal MSNs, where the long-term consequence could be mitochondrial dysfunction and axon degeneration in striatum over time^71^. We previously showed that *Actn2* mRNA levels are reduced in human HD caudate providing validity to our observations^51^. The functional consequences of the reduced striatal *Actn2* expression levels in YAC128 mice thus prompt a more detailed investigation.

There is ample evidence that BAX contributes to HD pathogenesis with evidence suggesting that mHTT causes accumulation and translocation of BAX to the outer mitochondrial membrane in neurons^74^. BAX dysregulation in HD may not be brain specific as BAX protein levels were also higher in lymphocytes and monocytes from HD patients^75^. Interestingly, we showed here that *Bax* transcript levels were increased in YAC128 mice at 1, 9 and 21 months of age possibly suggesting that pro-apoptotic processes are initiated early in skeletal muscle of HD mice^76^. Our finding therefore warrants further investigation of the consequences of the dysregulation of *Bax* in skeletal muscle where future studies should aim to go beyond analyzing the mRNA and protein levels of the members in the Bcl-2 family as they might not correlate with protein activity, especially given that most BH3-only members undergo strong regulation by post-translational mechanisms. In this study, we also report transcript levels of genes involved in the fusion and fission machinery. While we found age-related transcriptional changes in *Mfn1, Mfn2, Opa1* and *Drp1*, we found no genotype effects. This contrasts with previous studies on human HD brain where increased *Drp1* and *Fis1* and decreased *Mfn1*, *Mfn2* and *Opa1* expression levels were reported^77^. The reason for these discrepancies is not clear, but might be related to methodological differences (e.g. quantification, normalization, sample size) or that mHTT in these mice does not affect the transcript levels of these genes. Furthermore, mtDNAcn is regulated by the membrane fusion machineries^78^. Our findings indicated no overt correlation between the transcript levels of the fusion factors and mtDNAcn, suggesting that these factors may be regulated through post-translational modifications^79^ or that other regulatory factors affect the mtDNA content in YAC128 skeletal muscle. All things considered, future studies will explore the exact mechanisms underlying the altered mtDNAcn in cortex and skeletal muscle of YAC128 mice.

mtDNA deletions and mutations have been reported to accumulate with age with most studies showing that the overall fraction of Δ-mtDNA is low, while others have shown high fractions and higher degree of Δ-mtDNA in energy-dependent tissues such as brain and skeletal muscle^34^ with the highest levels observed in putamen, substantia nigra and cerebral cortex^80^. Moreover, mtDNA deletions have been reported in Parkinson’s disease (PD)^32,81^ and a causative link between mtDNA mutations and aging phenotypes has been established in mammals^82^. Interestingly, we detected mtDNA deletions in mice already at 1 month of age in striatum. The relatively high prevalence of mtDNA deletions in young mice is a surprising finding suggesting that mtDNA in striatum is more vulnerable to damage than in skeletal muscle. This finding is in agreement with previous studies showing increased mtDNA damage in striatum compared to other brain regions in R6/2 and WT mice^28^. Hence, data from this and other studies converge to indicate that mtDNA in striatum is vulnerable to damage, although not providing a sufficient explanation for the specific vulnerability of the striatum in HD. Because mtDNA is spatially close to the source of reactive oxygen species (ROS), it is assumed to be particularly vulnerable to ROS-mediated mutations^83,84^, although studies have provided evidence for that aging phenotypes generated by the accumulation of mtDNA damage are not mediated by increased ROS production^33^. Regardless of the underlying reason, our data suggest increased vulnerability to or exposure to factors causing mtDNA damage in the striatum. One limitation of the deletion analysis is the number of samples analyzed in combination with the detection method having relatively low sensitivity. It was further done on tissue homogenate that generally identifies low levels of mtDNA deletions, in comparison to single-cell-based approaches^34^. Hence, these data need to be replicated in larger sample size and preferably with a more sensitive detection method. It is further possible that point mutations with functional consequences exist in the mtDNA and those would not have been detected here.

In recent years, efforts have been made attempting to develop therapeutic strategies targeting mitochondria in neurodegenerative disease including HD^31,85^. Additionally, identification and implementation of biomarkers in HD are needed to support both pre-clinical research and human trials as efficacy and prognostic biomarkers are lacking. Identifying biomarkers that could be assessed in peripheral tissue such as skeletal muscle would thus potentially facilitate the process of drug development for HD. Interestingly, reduced mtDNAcn have been shown in peripheral leukocytes from HD patients with increased levels before the onset of motor symptoms and reduced levels in symptomatic HD patients, suggesting this as a viable biomarker^86^. Recent studies have further reported a concordant reduction in mtDNAcn in substantia nigra pars compacta tissue and in peripheral blood from PD patients^87^. Interestingly, treatment with the pan-PPAR agonist Bezafibrate ameliorated the clinical symptoms in the R6/2 and BACHD mice including reversing the muscle fiber switch back to levels observed in the WT mice (type II to type I fibers), while increasing the numbers of mitochondria in both muscle and striatum^88,89^. Yet, another study showed beneficial effects of the Drp1/Fis inhibitor, P110, leading to improved phenotype and increased mtDNA levels in brain and plasma of R6/2 mice and in plasma from HD patients^90^. Although the utility of measuring mtDNAcn in brain is limited, its prospects as a potential biomarker in HD skeletal muscle should be further examined.

## Methods

### Animals and ethics

All of the experiments were performed on the YAC128 mouse model of HD maintained on the FVB/N strain background^36^. Mice were group-housed with littermates in polystyrene cages under a normal light−dark cycle (6 am to 8 pm) in a clean facility and with free access to water and standard rodent chow. All experiments were performed in accordance with protocols approved by the University of British Columbia Animal Care Committee (ACC).

### Tissue collection

Animals were anesthetized using Avertine and striatum, pre-frontal cortex and quadriceps skeletal muscle tissue were then immediately collected. Striatal tissue was harvested in RNAlater® (Ambion) and then stored at −80 °C prior to DNA and RNA isolation. Cortical and skeletal muscle tissue was snap-frozen in liquid nitrogen upon collection and then stored at −80 °C.

### DNA and RNA isolation

Total RNA and DNA from mouse striatum and cortex were extracted using the Allprep DNA/RNA (Qiagen, Germany) mini kit according to the manufacturer’s instructions. Homogenization of tissue was performed using a Fastprep Homogenizer (ThermoScientific). Total RNA from skeletal muscle (quadriceps) was extracted with TRIzol™ Reagent (Invitrogen) according to the manufacturer’s protocol. To improve RNA purity following TRIzol extraction, muscle RNA was cleaned up in a subsequent step with the PureLink™ Mini Kit (Invitrogen) using an on-column-based approach. DNA from skeletal muscle was extracted using the DNeasy® kit (Qiagen, Germany) according to the manufacturer’s protocol. Total RNA and DNA was quantified using the Nanodrop™ spectrophotometer (ThermoScientific).

### Mitochondrial DNA quantification

For each DNA sample, the nuclear gene for the mouse polymerase gamma 2 accessory subunit (*Polg2*) and the mtDNA-encoded mouse cytochrome c oxidase 1 (*mt-Co1*) were amplified separately in duplicate by real-time quantitative polymerase chain reaction (qPCR) on a Lightcycler® 480 (Roche Diagnostics, USA) by use of the Lightcycler® 480 Probes master mix (Roche Diagnostics, USA). The PCR conditions were as follows: 95 °C for 10 min followed by 45 cycles of 95 °C for 5 s, 60 °C for 10 s, and 72 °C for 5 s. Absolute quantification was applied with standard curves prepared (1:10 serial dilutions) using plasmids with the gene cloned into the TOPO plasmid vector (pCR^@^2.1-TOPO^@^, Invitrogen, California), ranging from 4.63 × 10^1^ to 4.63 × 10^6^ copies for *Polg2* and from 2.09 × 10^1^ to 2.09 × 10^6^ copies for *mt-Co1*. A standard curve for each gene was included on every plate. mtDNA levels were expressed as the ratio of the mean value of the duplicate *mt-Co1* measurements to the mean value of duplicate *Polg2* measurements. For the nuclear gene (*Polg2)*, the forward primer Polg2-F (5ʹ-GGAGGAGGCACTTTCTCAGC-3ʹ) and reverse primer Polg2-R: (5ʹ-GAAGACCTGCTCCCTGAACAC-3ʹ) were used. The oligonucleotides 3ʹfluorescein Polg2PR1 (5ʹ-GCGCTTTGGACCTTTGGGTGTAG-F3ʹ) and 5′-LC Red 640 3′-phosphate-blocked Polg2PR2 (5ʹL-GTTACGAAAGAACCTAGCCTCACAGTGGT-P3ʹ) were used as hybridization probes. For the mitochondrial (*mt-Co1*) gene, the forward primer mt-Co1-F (5ʹ-TCGTTGATTATTCTCAACCAATCA-3ʹ) and mt-Co1-R (5ʹ-GCCTCCAATTATTATTGGTATTACTATGA-3ʹ) were used. The oligonucleotides 3ʹfluorescein mt-Co1PR1 (5ʹ-AACCAGGTGCACTTTTAGGAGATGACC-F3ʹ) and 5ʹ-LC Red 640 3ʹ phosphate-blocked mt-Co1PR2 (5ʹL-AATTTACAATGTTATCGTAACTGCCCATGC-P3ʹ) were used as hybridization probes.

### mtDNA deletion analysis

Long template PCR amplification was performed using Expand™ Long Range dNTPack (Roche Diagnostics, USA) on a MyCycler™ Thermal cycler (Bio-Rad, USA). Two individual PCR reactions were run to amplify two DNA fragments encompassing the entire mitochondrial genome. The following primers were used for PCR product 1 and PCR product 2, respectively: Long-PCR1-forward: 5ʹ-GCCCATACGTTCCCCTTAAATAAGAC-3ʹ; Long-PCR1-reverse:

5ʹ-TGTTGATGTATCTAGTTGTGGCATATCAC-3ʹ; Long-PCR2-forward: 5ʹ-CTGCTAGAAGTTGATAACCGAGTCGT-3ʹ; Long-PCR2-reverse:

5ʹ-ATGGAGGTTTGCATGTGTAATTTTACCTC-3ʹ. The fragments were separated by size on agarose gels. Gels were then digitized using the UN-Scan-IT software and the mtDNA deletion score was semi-quantified. The mtDNA deletion score was expressed as total deletion burden (%) and calculated as the ratio between deleted mtDNA over the total amount of mtDNA.

### Gene expression analysis

RNA samples were first treated with DNase I (Roche Diagnostics, USA) to eliminate genomic contamination and reverse transcription of RNA was then performed using the Superscript VILO cDNA Synthesis Kit (Invitrogen) according to the manufacturer’s instructions. Quantitative analyses of mRNA expression were performed using the Lightcycler® 480 SYBR green I master mix (Roche Diagnostics, USA) according to the manufacturer’s instructions. Amplification of cDNA was performed using the Lightcycler® 480 system (Roche Diagnostics, USA). All primers were designed to span exon/exon boundaries to avoid amplification of contaminating genomic DNA using the Primer Express software version 3.0 (Applied Biosystems) (Supplementary Table 7). Relative quantification of mRNA levels was calculated using the standard curve method and calculated as the ratio between the target mRNA and a normalization factor, that is a factor calculated from the endogenous levels of the reference genes analyzed. *Hprt1*, *Actb* and *Rplp0* were used to calculate the normalization factor (NF3) in both striatal and skeletal muscle tissue.

### Histological cytochrome c oxidase staining and analysis

Skeletal muscle tissue (quadriceps) and striatum were collected and put into 30% sucrose for cryo-protection and then put into optimal cutting temperature (OCT) compound and cut in Cryostate Microm 550 (Microm, Germany). Skeletal muscle (12 μm) and striatal tissues (25 μm) were cut in sections and mounted on Superfrost microscope slides. COX reaction mixture was prepared (50 mM sodium phosphate pH 7.2, 10 mg/ml cytochrome c (oxidized), 10 mg/ml diaminobenzidine, 200 mg/ml sucrose, catalase, 1600 units/ml). Reaction was started by adding ~5 ml reaction media to each histology container containing frozen tissue slides. Sodium azide, which inhibits COX, catalase and peroxidase reactions, was added to slides representing a negative control. The slides were incubated at 37 °C for 40 min on a gently rocking platform in a humid incubator. The reaction was quenched by performing three brief serial rinses with distilled water. The sections were dehydrated using 70% (2 min), 95% (2 min) and 100% (2 min) ethanol. The sections were then cleared in xylene and cover-slipped using DePeX (DPX) mounting medium.

### Quantification of COX-staining intensities

Images were taken using Zeiss Axioplan 2 microscope with the coolsnap HQ camera (Photometrics) using a ×5 objective. The amount of COX staining was quantified from images, which were acquired using MetaMorph® software (Molecular Devices, USA). The intensity of COX in quadriceps muscle was determined using MetaMorph® software as the integrated intensity per muscle fiber normalized to area of muscle fiber in approximately 200 fibers per mouse. For percentage of low-intensity COX-stained fibers measurements, all fibers from one section were visualized under a microscope at ×20 objective and determined as having either a subtle COX stain therefore considered low, versus fibers which were darkly stained for COX therefore considered high-intensity COX-stained fibers. The number of low-intensity COX-stained fibers was divided by the total number of COX-stained fibers and expressed as percentage of low-intensity COX-stained fibers. For striatal COX measurements, every 24th section from Bregma 1.54 mm to −1.06 mm was stained for COX, and striatum in each section was contoured. Integrated intensity per section was determined using MetaMorph® software. The average total integrated intensity was calculated for four sections per slide and with two slides being analyzed per mouse (in total 8 sections per mouse). Background COX integrated intensity staining obtained from a negative control section (sodium azide was added to COX-staining procedure to inhibit COX activity) was subtracted from the integrated intensity in each of the analyzed sections.

### HPLC measurement of adenylate nucleotide levels

The mice were sacrificed and striatum, cortex and skeletal muscle tissue were immediately dissected and collected. The tissue was homogenized in cold 5% trichloroacetic acid (TCA) using a 26G syringe needle and kept on ice for 30 min. Samples were centrifuged at 14,000 rpm, 4 °C, for 10 min to remove denatured proteins and cell debris. The supernatant was transferred into borosilicate tubes and the TCA extract was extracted five times with diethylether. The samples were allowed to air-dry and were heated to 50 °C for 3–5 min for the diethylether to evaporate after the final extraction. Samples were stored at −80 °C until etheno derivatization and HPLC analysis which was performed as described previously^91^. ATP is not only produced through mitochondrial oxidative phosphorylation, but also through glycolysis and direct ADP phosphorylation by adenylate kinase. In our experimental setting using HPLC analysis, we were unable to account for or eliminate the contribution of ATP from these other sources, although they are considered minor contributors to the ATP production.

### Transmission electron microscopy preparation and analysis

Striatal tissues from mice were analyzed at the ultrastructural level using TEM. YAC128 and WT mice at 1 (young) and 21–23 (old) months of age were analyzed (*n* = 3 per group). Prior to collection, each mouse was injected with 1000 USP units of heparin. Mice were then perfused for 30 min first with cardiac PBS and then with 3% paraformaldehyde (PFA) before collection of tissues. Tissues were stored overnight at 4 °C in PFA solution before processing for TEM. The tissue from each site was dehydrated, infiltrated and embedded in Epon. Three Epon-embedded blocks were randomly selected from each animal and sectioned at 60 nm using the Leica EM UC6 ultramicrotome (Leica Microsystems, Switzerland). The sections were stained with uranyl acetate and Sato’s lead for viewing under the FEI Tecnai 12 TEM equipped with a digital camera. For each block, five cell bodies were randomly selected and photographed at ×5800. The dendrite/axon regions adjacent to these cell bodies were also photographed at ×13,500. Higher magnification images of four quadrants of each cell body at N, E, S and W were obtained at ×46,000. All morphometric analyses were performed on the digital images using Image-Pro Plus 4.0 software (Media Cybernetics, Silver Spring, MD, USA). Volume fractions of mitochondria, lysosomes, Golgi and nuclei within cell bodies were determined by point counting, counting 15 cell bodies or axon/dendrite sites per mouse (in total 45 cell bodies or axon/dendrite sites group)^92^. The same technique was used to determine mitochondrial volume fractions in the axon/dendrite regions adjacent to the cell bodies. The line intercept technique using a linear grid was used to determine the surface area to volume ratios of ER and mitochondrial cristae within striatal cell bodies^92^.

### Statistical analysis

Statistical analyses were done in Stata (version 16) and figures were generated using JMP (version 14). mtDNAcn and gene expression data were analyzed using linear regression and cubical spline regression analysis including host factors (age, sex and genotypes as fixed factors). Regarding the model selection, both linear and spline regression analyses were performed for each test and the most appropriate model was selected based on the line of best fit. In cubic spline regression, time was modeled using restricted randomized cubic splines^93^, connected by knots and constricted to follow a linear distribution before the first and after the last knot. Splines were randomized and fitted to specified knots. To investigate the genotype-specific effects, we run the above specific analysis for each genotype separately. Dynamics of mtDNAcn was further investigated using multivariate regression analysis, fitting age as a categorical variable. Comparison of the age groups and genotype was further explored using Tukey’s post-hoc multiple comparison test. The test for normality, Shapiro-Francia, indicated the different data sets were statistically normal, except for the Δ-mtDNA data set. Binomial generalized linear models were used for analysis of the Δ-mtDNA data set (dependent variable, 0 = no Δ-mtDNA or 1 = Δ-mtDNA). Unpaired two-sided *t* tests were used to analyze the difference between two groups at specific time points. Two-way analysis of variance (ANOVA) with Tukey’s post-hoc multiple comparison test was used to examine the influence of two categorical independent variables (age and genotype) on continuous dependent variables (Graph Pad Prism version 7.0). Data are reported as mean ± s.e.m or box and whisker plots showing the median with the box extending from the 25th to the 75th percentile, and the whiskers showing the smallest value up to the largest. *p* values < 0.05 were considered significant.

### Reporting summary

Further information on research design is available in the Nature Research Reporting Summary linked to this article.

## Supplementary information

Supplementary Information

Reporting Summary

## Data Availability

The data that supports the findings of this study are available from the corresponding author upon reasonable request.
