# Author Correction: Age-related mitochondrial alterations in brain and skeletal muscle of the YAC128 model of Huntington disease

**DOI:** 10.1038/s41514-021-00080-9

**Published:** 2021-12-07

**Authors:** Kristina Bečanović, Muhammad Asghar, Izabella Gadawska, Shiny Sachdeva, David Walker, Eduardo. R. Lazarowski, Sonia Franciosi, Kevin H. J. Park, Hélène C. F. Côté, Blair R. Leavitt

**Affiliations:** 1 Centre for Molecular Medicine and Therapeutics, Department of Medical Genetics, University of British Columbia, Vancouver, BC Canada; 2 Department of Clinical Neuroscience, Karolinska Institutet, Stockholm, Sweden; 3 Department of Medicine, Division of Infectious Diseases, Karolinska Institutet, Stockholm, Sweden; 4 Department of Infectious Diseases, Karolinska University Hospital, Stockholm, Sweden; 5 Department of Pathology & Laboratory Medicine, University of British Columbia, Vancouver, BC Canada; 6 The James Hogg iCAPTURE Centre for Cardiovascular and Pulmonary Disease, St Paul’s Hospital, Vancouver, BC Canada; 7 Cystic Fibrosis Research Center, Marsico Lung Institute, University of North Carolina, Chapel Hill, NC USA; 8 Department of Pediatrics, University of British Columbia, Vancouver, BC Canada; 9 Department of Psychology and Neuroscience Program, Central Michigan University, Mount Pleasant, MI USA

**Keywords:** Ageing, Neurodegeneration

Correction to: *npj Aging and Mechanisms of Disease* 10.1038/s41514-021-00079-2, published online 14 October 2021

In the original version of this Article, Muhammad Asghar was mistakenly referred to as Asghar Muhammad. This has now been corrected in both the PDF and HTML versions of the Article.

