# Supplementary material for: Age-related mitochondrial alterations in brain and skeletal muscle of the YAC128 model of Huntington disease
Source: NPJ Aging Mech Dis. 2021 Oct 14;7:26. doi: 10.1038/s41514-021-00079-2 (PMC8516942; doi:10.1038/s41514-021-00079-2)
Supplement: Supplementary file 1 — Supplementary Information [file 41514_2021_79_MOESM1_ESM.pdf]

Supplementary Table 1

Effects of age and sex on mtDNA copy number in striatum, cortex and skeletal muscle of YAC128 and WT mice

Tissue	Variables	Spline[3]	Coef.	Std.Err.	DF	t-value	p-value	95% CI[4]
Striatum								
WT[1]	Age		22.1	6.21	52	3.56	0.001	9.63 – 34.6
	Sex		-71.5	86.1	52	-0.83	0.411	-244.5 – 101.6
YAC128[2]	Age Spline 1	1-6	75.9	62.8	69	1.21	0.231	-49.4 – 201.4
	Age Spline 2	6-12	-565.0	334.3	69	-1.69	0.096	-1,232.8 – 102.8
	Age Spline 3	12-18	1,938	888.4	69	2.18	0.033	163.0 – 3,713
	Age Spline 4	18-21	-3,568	1,196.7	69	-2,98	0.004	5,958 – 1,177
	Sex		6.31	108.6	69	0.06	0.954	-210.6 – 223.2
Cortex								
WT[2]	Age Spline 1	1-6	67.6	31.7	60	2.13	0.037	4.16 – 131.1
	Age Spline 2	6-21	-150.2	78.0	60	-1.94	0.058	-307.4 – 5.00
	Sex		-100.5	135.4	60	-0.74	0.461	-371.7 – 170.6
YAC128[2]	Age Spline 1	1-6	86.7	44.8	81	1.94	0.057	-2.51 – 175.8
	Age Spline 2	6-12	-574.5	251.9	81	-2.28	0.025	-1,076 – 72.6
	Age Spline 3	12-18	1,868	693.8	81	2.69	0.009	485.7 – 3,249
	Age Spline 4	18-21	-3,459	1,001	81	-3.45	0.001	-5,453 – -1,465
	Sex		65.5	91.1	81	0.72	0.474	-115.8 – 246.9
Skeletal muscle								
WT[1]	Age		448.6	139.5	64	3.21	0.002	169.7 – 727.5
	Sex		904.2	2,024.7	64	0.45	0.657	-3,143 – 4,942
YAC128[2]	Age Spline 1	1-9	1,104	355.0	78	3.11	0.003	397.8 – 1,812
	Age Spline 2	9-21	-1,473	533.3	78	-2.76	0.007	-2,535 – -410.5
	Sex		370.7	1,611	78	0.23	0.819	-2,839 – 3,580

1) Linear regression was used for analysis of mtDNA copy number in striatum and skeletal muscle of WT mice.
2) Cubic spline regression was used for analysis of mtDNA copy number in striatum of YAC128, cortex of WT and YAC128 mice, and skeletal muscle of YAC128 mice, respectively.
3) Spline indicates the knots in the cubic spline regression.
4) 95% confidence interval

Supplementary Table 2[1]
Significant differences in mtDNA copy number in YAC128 compared to WT mice at specific ages

Tissue	Age (months)	Mean difference[2]	Std.Err.	DF	t-value	p-value
Striatum	12	-342.8	135.2	18	-2.54	0.021
Cortex	6	-506.9	228.7	14	-2.22	0.044
	9	-365.7	161.4	9	-2.27	0.049
	12	-648.8	220	18	-2.95	0.008
Skeletal muscle	3	-6,120	2,784	18	-2.20	0.041
	21	-7,158	3,048	20	-2.35	0.029

1) This table shows the statistical results for the age comparisons that were statistically significant using pooled t-test ($p<0.05$).
2) Negative values reflect lower mean mtDNA copy number in YAC128 compared to WT mice.

Supplementary Table 3
Average mtDNA copy number (mtDNAcn) in striatum, cortex and skeletal muscle of YAC128 and WT mice

Age (months)	Average mtDNAcn in striatum					
	WT	n[1]	Sex[2] (M/F)	YAC128	n[1]	Sex[2] (M/F)
1	1,197	14	4/10	1,297	9	2/7
3	1,272	9	3/6	1,214	11	6/5
6	1,172	1	0/1	1,436	5	3/2
9	1,335	5	2/3	1,449	6	1/5
12	1,372	10	5/5	1,029	10	4/6
15	1,459	5	3/2	1,818	10	4/6
18	1,651	5	4/1	1,896	7	4/3
21	1,721	4	1/3	1,609	12	5/7

Age (months)	Average mtDNAcn in cortex					
	WT	n[1]	Sex[2] (M/F)	YAC128	n[1]	Sex[2] (M/F)
1	1,623	18	7/11	1,378	15	7/8
3	1,335	8	2/6	1,442	11	6/5
6	2,236	6	3/3	1,730	10	6/4
9	1,673	5	2/3	1,308	6	1/5
12	1,996	10	5/5	1,348	10	4/6
15	1,641	5	3/2	2,006	10	4/6
18	1,947	5	4/1	1,769	8	5/3
21	1,492	4	1/3	1,425	12	5/7

Age (months)	Average mtDNAcn in skeletal muscle					
	WT	n[1]	Sex[2] (M/F)	YAC128	n[1]	Sex[2] (M/F)
1	10,487	17	7/10	8,215	14	7/7
3	15,882	9	3/6	9,762	11	6/5
6	16,349	6	3/3	18,963	10	6/4
9	18,511	5	2/3	18,996	6	1/5
12	14,580	10	5/5	14,138	10	4/6
15	20,748	4	3/1	13,839	8	4/4
18	15,893	5	4/1	10,160	7	4/3
21	21,880	9	8/1	14,722	13	6/7

1) Total number of mice analyzed.
2) Number of male mice (M) and female mice (F)

Supplementary Table 4
Effects of age, genotype and sex on gene expression in striatum and skeletal muscle of YAC128 and WT mice

Gene/ Tissue[3]	Variables	Spline	Coef.	Std.Err.	t-value	DF	p-value	95% CI
Pgc-1α								
STR[1]	Age		-1.49	6.92	-0.22	88	0.830	-15.3 – 12.3
	Sex		-68.6	109.4	-0.63	88	0.532	-286.1 – 148.9
	Genotype		-120.7	109.3	-1.10	88	0.273	-338.1 – 96.6
SKM[2]	Age Spline 1	1-3	183.1	79.6	2.30	104	0.024	25.1 – 341.0
	Age Spline 2	3-21	-842.2	417.6	-2.02	104	0.046	-1,671 – -13.7
	Sex		-241.5	281.0	-0.86	104	0.392	-799.1 – 316.0
	Genotype		219.9	272.8	0.81	104	0.422	-321.3 – 761.0
Pparδ								
STR[1]	Age		-3.85	7.72	-0.50	89	0.619	-19.2 – 11.5
	Sex		13.9	122.3	0.11	89	0.910	-229.2 – 256.9
	Genotype		-34.7	122.6	-0.28	89	0.778	-278.4 – 208.9
SKM[2]	Age Spline 1	1-3	168.4	129.6	1.30	109	0.197	-88.6 – 425.4
	Age Spline 2	3-12	-1,782	1,449	-1.23	109	0.222	-4,656 – 1,091
	Age Spline 3	12-21	2,323	1,893	1.23	109	0.223	-1,431 – 6,077
	Sex		-82.4	179.9	-0.46	109	0.648	-439.1 – 274.3
	Genotype		118.1	178.3	0.66	109	0.509	-235.5 – 471.8
p62								
STR[2]	Age Spline 1[d]	1-12	409.9	103.3	3.97	88	<0.0001	204.5 – 615.4
	Age Spline 2[e]	12-21	-467.7	160.0	-2.92	88	0.004	-785.9 – -149.5
	Sex		-317.5	464.7	-0.68	88	0.496	-1,242 – 606.6
	Genotype		526.6	464.5	1.13	88	0.260	-397.1 – 1,450.3
SKM[2]	Age Spline 1	1-3	3,217	570.5	5.64	109	<0.0001	2,085 – 4,348
	Age Spline 2	3-9	-43,854	8,466	-5.18	109	<0.0001	-60,644 – -27,063
	Age Spline 3	9-18	65,123	12,449	5.23	109	<0.0001	40,432 – 89,814
	Age Spline 4	18-21	-30,455	5,737	-5.31	109	<0.0001	-41,838 – 19,078
	Sex		-1,883	681.2	-2.76	109	0.007	-3,234 – -531.8
	Genotype		-1,076	668.7	-1.61	109	0.110	-2,402 – 249.6
LC3b								
STR[2]	Age Spline 1	1-9	-349.4	84.0	-4.16	89	<0.0001	-516.4 – -182.4
	Age Spline 2	9-21	461.3	129.6	3.56	89	0.001	203.6 – 719
	Sex		-224.8	376.5	-0.60	89	0.552	-973.3 – 523.7
	Genotype		-495.4	378.0	-1.31	89	0.194	-1247 – 256.1
SKM[2]	Age Spline 1	1-3	2,466	522.2	4.72	107	<0.0001	1,430.5 – 3,502
	Age Spline 2	3-9	-29,170	6,980.2	-4.18	107	<0.0001	43,015 – 15,325
	Age Spline 3	9-21	40,765	9,829.9	4.15	107	<0.0001	21,268 – 60,263
	Sex		100.6	652.9	0.15	107	0.878	-1,194 – 1,396
	Genotype		-234.9	638.7	-0.37	107	0.714	-1,502 – 1,032
Bax								
STR[2]	Age Spline 1	1-9	-36.2	6.87	-5.26	89	<0.0001	-49.8 – 22.5
	Age Spline 2	9-18	92.2	19.6	4.70	89	<0.0001	53.2 – 131.2
	Age Spline 3	18-21	-308.1	68.8	-4.48	89	<0.0001	-444.9 – 171.3
	Sex		52.3	22.4	2.33	89	0.022	7.70 – 96.9
	Genotype		4.26	22.1	0.19	89	0.848	-39.6 – 48.2
SKM[2]	Age Spline 1	1-3	40.5	11.24	3.60	104	<0.0001	18.2 – 62.8
	Age Spline 2	3-9	-639.9	149.9	-4.27	104	<0.0001	-937.4 – -342.5
	Age Spline 3	9-21	914.9	211.0	4.34	104	<0.0001	496.2 – 1,334
	Sex		-7.24	13.96	-0.52	104	0.605	-34.9 – 20.5
	Genotype		29.7	13.60	2.18	104	0.031	2.69 – 56.7
Bcl-XL								
STR[2]	Age Spline 1	1-3	-320.8	122.8	-2.61	89	0.011	12.1 – 70.7
	Age Spline 2	3-18	3,143	1,198	2.62	89	0.010	-1,160 – -231.2
	Age Spline 3	18-21	-3,848	1,500	-2.56	89	0.012	-741.7 – 189.2
	Sex		-639.8	227.3	-2.81	89	0.006	-1,091 – -187.7
	Genotype		-251.1	226.5	-1.11	89	0.271	-701.6 – 199.4
SKM[1]	Age	107	-0.549	4.57	-0.12	107	0.904	-9.61 – 8.51
	Sex	107	-130.8	71.7	-1.82	107	0.071	-272.9 – 11.5
	Genotype	107	78.6	70.4	1.12	107	0.266	-60.9 – 218.2

Drp1								
STR[2]	Age Spline 1	1-3	-1,344	436.7	-3.08	89	0.003	-2,213 – -475.7
	Age Spline 2	3-12	17,257	5,060	3.41	89	0.001	7,195 – 27,319
	Age Spline 3	12-21	-22,839	6,656	-3.43	89	0.001	-36,075 – 9,602
	Sex		-881.9	629.2	-1.40	89	0.165	-2,133 – 369.2
	Genotype		-192.9	633.6	-0.30	89	0.761	-1,453 – 1,067
SKM[2]	Age Spline 1	1-3	703.2	156.5	4.49	109	<0.0001	392.8 – 101.4
	Age Spline 2	3-9	-14,694	2,323	-6.33	109	<0.0001	-19,300 – 10,087
	Age Spline 3	9-18	22,789	3,416	6.67	109	<0.0001	16,015 – 29,563
	Age Spline 4	18-21	-12,206	1,574	-7.76	109	<0.0001	-15,327 – 9,085
	Sex		-485.5	186.9	-2.60	109	0.011	-856.2 – -114.9
	Genotype		138.4	183.4	0.75	109	0.452	-225.4 – 502.3
Mfn1								
STR[1]	Age	87	10.6	4.01	2.63	87	0.010	2.6 – 18.5
	Sex	87	-60.5	63.5	-0.95	87	0.343	-186.9 – 65.8
	Genotype	87	-16.3	63.2	-0.26	87	0.797	-142.0 – 109.5
SKM[2]	Age Spline 1	1-3	1102.8	259.2	4.26	109	<0.0001	588.9 – 1,617
	Age Spline 2	3-12	-12,184	2,898	-4.20	109	<0.0001	-17,932 – -6,438
	Age Spline 3	12-21	15,876	3,786	4.19	109	<0.0001	8,369 – 23,383
	Sex		-644.1	359.7	-1.79	109	0.076	-1,357 – 69.1
	Genotype		-124.0	356.6	-0.35	109	0.729	-831.1 –583.1
Mfn2								
STR[1]	Age	89	-172.9	38.8	-4.45	89	<0.0001	-250.0 – -95.7
	Sex	89	-111.3	614.9	-0.18	89	0.857	-1,334 – 1111
	Genotype	89	-710.9	616.5	-1.15	89	0.252	1,937 – 514.7
SKM[2]	Age Spline 1	1-3	2,253	908.7	2.48	109	0.015	451.0 – 4,055
	Age Spline 2	3-9	-27,706	12,086	-2.29	109	0.024	-51,674 – 3,737
	Age Spline 3	9-21	38,990	17,001	2.29	109	0.024	5,276 – 72,705
	Sex		-1,588	1,119	-1.42	109	0.159	-3,807 – 631.4
	Genotype		-576.9	1,111	-0.52	109	0.605	-2,781 – 1,627
Opa1								
STR[2]	Age Spline 1	1-9	-384.0	117.7	-3.26	89	0.002	-618.1 – -149.9
	Age Spline 2	9-12	1,372	438.5	3.13	89	0.002	500.4 – 2,245
	Age Spline 3	12-21	-8,519	2,668	-3.19	89	0.002	-13,826 – -3,213
	Sex		130.6	286.0	0.46	89	0.649	-438.1 – 699.4
	Genotype		-120.0	284.2	-0.42	89	0.674	-685.2 – 445.2
SKM[2]	Age Spline 1	1-3	641.9	237.1	2.71	109	0.008	171.7 – 1,112
	Age Spline 2	3-9	-9,680	3,154	-3.07	109	0.003	-15,935 – -3,425
	Age Spline 3	9-21	13,729	4,437	3.09	109	0.003	4,931 – 22,527
	Sex		-361.1	292.0	-1.24	109	0.219	-940.2 – 217.9
	Genotype		-287.7	290.0	-0.99	109	0.324	-862.9 –287.4
mt-Co1								
STR[1]	Age	88	299.7	173.8	1.72	88	0.088	-45.9 – 645.3
	Sex	88	-5,935.2	2,766.1	-2.15	88	0.035	-11,435 – -435.4
	Genotype	88	200.7	2,774.1	0.07	88	0.942	-5,316 – 5,717
SKM[2]	Age Spline 1	1-3	12,585	6,149	2.05	109	0.043	392.0 – 24,788
	Age Spline 2	3-9	-168,417	81,782	-2.06	109	0.042	-330,595 – -6,239
	Age Spline 3	9-21	237,198	115,035	2.06	109	0.042	9,078 – 465,317
	Sex		-24,646	7,571	-3.26	109	0.002	-39,661 – -9,631
	Genotype		-531.7	7,520	-0.07	109	0.944	-15,444 – 14,380
Actn2								
STR[1]	Age	89	-273.4	45.7	-5.98	89	<0.0001	-364.3 – -182.5
	Sex	89	1,122.6	724.6	1.55	89	0.125	-317.8 – 2,563
	Genotype	89	-9,154.9	726.5	-12.6	89	<0.0001	-10,599 – -7,711
SKM[2]	Age Spline 1	1-3	18,602	7,049	2.64	109	0.010	4,623 – 32,581
	Age Spline 2	3-12	-258,235	78,832	-3.28	109	0.001	-414,563 – -101,907
	Age Spline 3	12-21	343,408	102,980	3.33	109	0.001	139,194 – 547,622
	Sex		-10,499	9,784	-1.07	109	0.286	-29,900 – 8,903
	Genotype		12,132	9,700	1.25	109	0.214	-7,103 – 31,366

1) Linear regression was used for analysis.
2) Cubic spline regression was used for analysis.
3) Genes are indicated in italics and bold text. Tissue: STR=Striatum; SKM= Skeletal muscle

Supplementary Table 5[1]
Difference in gene expression levels in YAC128 compared to WT mice at specific ages

Gene[2]	Tissue[3]	Age (months)	Mean difference[4]	Std.Err.	DF	t-value	p-value
p62	STR	21	-2,941	878.5	13	-3.34	0.0053
	SKM	21	-2,826	1,321	18	-2.14	0.046
Bax	SKM	1	47.9	17.8	26	2.69	0.012
	SKM	9	66.0	28.6	8	2.31	0.049
	SKM	21	66.9	27.2	18	2.46	0.024
mt-Co1	STR	9	8,865	3,373	8	2.63	0.030
Actn2	STR	1	-8,930	1,395	22	-6.40	<0.0001
	STR	3	-9,774	1,168	17	-8.37	<0.0001
	STR	9	-9,576	2,167	9	-4.41	0.017
	STR	12	-7,557	1,791	5	-4.21	0.0083
	STR	18	-7,478	1,914	11	-3.91	0.0024
	STR	21	-10,818	2,721	14	-3.98	0.0014
	SKM	18	52,420	20,269	10	2.59	0.027
	SKM	21	39,981	15,967	18	2.50	0.022

1) Table shows the results for the age comparisons that were statistically significant using pooled t-test ($p<0.05$).
2) The genes are indicated in italics.
3) STR=Striatum; SKM=Skeletal muscle
4) Negative values reflect lower mean transcript mRNA levels in YAC128 compared to WT.

Supplementary Table 6
Number of mice analyzed in gene expression analysis

Age (months)	STRIATUM				SKELETAL MUSCLE			
	WT	Sex[1] (M/F)	YAC128	Sex[1] (M/F)	WT	Sex[1] (M/F)	YAC128	Sex[1] (M/F)
1	13	4/9	11	4/7	16	7/9	14	7/7
3	8	2/6	11	6/5	9	3/6	11	6/5
9	5	2/3	6	1/5	4	2/2	6	1/5
12	4	2/2	3	2/1	9	5/4	7	4/3
18	5	4/1	8	5/3	5	4/1	7	4/3
21	4	1/3	12	5/7	9	8/1	13	6/7

1) Number of male mice (M) and female mice (F)

Supplementary Table 7
Primers used in gene expression analysis (quantitative real-time PCR)

Gene target	Forward primer nucleotide sequence	Reverse primer nucleotide sequence	Amplicon size (basepairs)
mt-Co1	TCGTTGATTATTCTCAACCAATCA	GCCTCCAATTATTATTGGTATTACTATGA	220
Pgc-1a	GCGTCATTCGGGAGCTGG	CGCTACACCACTTCAATCCAC C	190
Pparδ	CGAGTTTGCTGTCAAGTTCAATG	TGGCCGGTCTCCACACA	91
LC3b	CTTTGAACAAAGAGTGGAAGA TGTCCG	ACCATGCTGTGCCCATTCACCA	231
p62	CACAGGAAGGACCCACAGGG	GAGCAGCCCCGATGTCGTAA	174
Bax	TGCACTAAAGTGCCCGAGCTG	ACACAGTCCAAGGCAGTGGG	229
Bcl-XL	AAACTGGGGTCGCATCGTGG	CTCTCGGCTGCTGCATTGTTCC	204
Drp1	TCAGCCCAAGGACATCGAGC	GGCATCAGTACCCGCATCCA	208
Mfn1	AATGCACAGAGGGTGCTGCT	GGAGGTTCTCGACGTGAGGG	171
Mfn2	CCCATGCCCTCCATCAGGAC	AGCGTGGACTCTGAGTTGGC	208
Opa1	CAGGTCACGCCAAAGCACTG	CCAGGCAACCTCGACTGC	195
Actn2	GCGCAGGAGCTCAATGAACT	CTATCCCACTGGTCGCAAATTT	83
Rplp0	TGGGCATCACCACGAAAAT	ATCAGCTGCACATCACTCAGAATT	61
Hprt1	CGTCGTGATTAGCGATGATGA	TCCAAATCCTCGGCATAATGA	78
Actb	CCAGCCTTCCTTCTTGGGTAT	TGTGTTGGCATAGAGGTCTTTACG	106
