# Supplementary material for: Age-related mitochondrial alterations in brain and skeletal muscle of the YAC128 model of Huntington disease
Source: NPJ Aging Mech Dis. 2021 Oct 14;7:26. doi: 10.1038/s41514-021-00079-2 (PMC8516942; doi:10.1038/s41514-021-00079-2)
Supplement: Supplementary file 2 — Reporting Summary [file 41514_2021_79_MOESM2_ESM.pdf]

nature research

Corresponding author(s): Kristina Becanovic

Last updated by author(s): Sep 2, 2021

Reporting Summary

Nature Research wishes to improve the reproducibility of the work that we publish. This form provides structure for consistency and transparency in reporting. For further information on Nature Research policies, see our Editorial Policies and the Editorial Policy Checklist.

Statistics

For all statistical analyses, confirm that the following items are present in the figure legend, table legend, main text, or Methods section.

n/a	Confirmed	
☐	☒	The exact sample size (n) for each experimental group/condition, given as a discrete number and unit of measurement
☐	☒	A statement on whether measurements were taken from distinct samples or whether the same sample was measured repeatedly
☐	☒	The statistical test(s) used AND whether they are one- or two-sided<br>*Only common tests should be described solely by name; describe more complex techniques in the Methods section.*
☐	☒	A description of all covariates tested
☐	☒	A description of any assumptions or corrections, such as tests of normality and adjustment for multiple comparisons
☐	☒	A full description of the statistical parameters including central tendency (e.g. means) or other basic estimates (e.g. regression coefficient) AND variation (e.g. standard deviation) or associated estimates of uncertainty (e.g. confidence intervals)
☐	☒	For null hypothesis testing, the test statistic (e.g. F, t, r) with confidence intervals, effect sizes, degrees of freedom and P value noted<br>*Give P values as exact values whenever suitable.*
☒	☐	For Bayesian analysis, information on the choice of priors and Markov chain Monte Carlo settings
☒	☐	For hierarchical and complex designs, identification of the appropriate level for tests and full reporting of outcomes
☒	☐	Estimates of effect sizes (e.g. Cohen's d, Pearson's r), indicating how they were calculated

Our web collection on statistics for biologists contains articles on many of the points above.

Software and code

Policy information about availability of computer code

Data collection	NA
Data analysis	NA

For manuscripts utilizing custom algorithms or software that are central to the research but not yet described in published literature, software must be made available to editors and reviewers. We strongly encourage code deposition in a community repository (e.g. GitHub). See the Nature Research guidelines for submitting code & software for further information.

Data

Policy information about availability of data

All manuscripts must include a data availability statement. This statement should provide the following information, where applicable:

- Accession codes, unique identifiers, or web links for publicly available datasets
- A list of figures that have associated raw data
- A description of any restrictions on data availability

The data that supports the findings of this study are available from the corresponding author upon reasonable request.

Field-specific reporting

Please select the one below that is the best fit for your research. If you are not sure, read the appropriate sections before making your selection.

☒ Life sciences ☐ Behavioural & social sciences ☐ Ecological, evolutionary & environmental sciences

For a reference copy of the document with all sections, see nature.com/documents/nr-reporting-summary-flat.pdf

Life sciences study design

All studies must disclose on these points even when the disclosure is negative.

Sample size	In the TEM analysis we analyzed 3 mice per group. Demonstration of significant quantitative differences among the phenotypes would have probably required a sample size of greater than five mice per phenotype. We performed two-way ANOVA and present the p-values and the mean +/-SEM in the results. We present the data in dot plots. For the mitochondrial DNA quantification analysis and gene expression analysis, we aimed to include five mice per age- and genotype group. There is some variability to this with a couple of groups which have fewer than 5 samples. We are however presenting the data in a transparent manner with scatter plots and the exact number included in the analysis are clearly indicated in the supplementary tables..
Data exclusions	Overall no data was excluded from the analysis except for on old YAC128 mouse from the transmission electron microscopy analysis due to a tumor in the brain.
Replication	Experiments were not replicated. However, we assayed the samples in duplicates where appropriate e.g. qPCR reactions.
Randomization	Where possible the experiments were designed such that both genotypes were represented on plates, slides etc. in a randomized manner.
Blinding	Blinding was not relevant to this study. The genotype of each mouse was double-checked. First genotype when born and second when mice were sacrificed.

Reporting for specific materials, systems and methods

We require information from authors about some types of materials, experimental systems and methods used in many studies. Here, indicate whether each material, system or method listed is relevant to your study. If you are not sure if a list item applies to your research, read the appropriate section before selecting a response.

Materials & experimental systems

n/a	Involved in the study	
☒	☐	Antibodies
☒	☐	Eukaryotic cell lines
☒	☐	Palaeontology and archaeology
☐	☒	Animals and other organisms
☒	☐	Human research participants
☒	☐	Clinical data
☒	☐	Dual use research of concern

Methods

n/a	Involved in the study	
☒	☐	ChIP-seq
☒	☐	Flow cytometry
☒	☐	MRI-based neuroimaging

Animals and other organisms

Policy information about studies involving animals; ARRIVE guidelines recommended for reporting animal research

Laboratory animals	All of the experiments were performed on the YAC128 mouse model of HD maintained on the FVB/N strain background and littermates. The age range was between 1-23 months of age. Both Female and male mice were assessed.
Wild animals	NA
Field-collected samples	NA
Ethics oversight	All experiments were performed in accordance with protocols approved by the University of British Columbia Animal Care Committee (ACC).

Note that full information on the approval of the study protocol must also be provided in the manuscript.
